# Lack of STAT1 co-operative DNA binding protects against adverse cardiac remodelling in acute myocardial infarction

**DOI:** 10.3389/fcvm.2023.975012

**Published:** 2023-02-27

**Authors:** Asmma Doudin, Theresa Riebeling, Julia Staab, Priyanka Rajeev Menon, Fred Lühder, Oliver Wirths, Uwe Vinkemeier, Aleksandar Ivetic, Thomas Meyer

**Affiliations:** ^1^Department of Psychosomatic Medicine and Psychotherapy, University Medical Centre Göttingen, and German Centre for Cardiovascular Research (DZHK), Partner Site Göttingen, Göttingen, Germany; ^2^Department of Nephrology and Hypertension, University Hospital Schleswig-Holstein, Kiel, Germany; ^3^Institute for Neuroimmunology and Multiple Sclerosis Research, University Medical Centre Göttingen, Göttingen, Germany; ^4^Department of Psychiatry and Psychotherapy, University Medical Centre Göttingen, Göttingen, Germany; ^5^Division of Infections, Immunity and Microbes, School of Life Sciences, University of Nottingham, Nottingham, United Kingdom; ^6^British Heart Foundation Centre, School of Cardiovascular and Metabolic Medicine and Sciences, King’s College London, London, United Kingdom

**Keywords:** STAT proteins, co-operative DNA binding, myocardial infarction, RNA sequencing, inflammation

## Abstract

In this study, we addressed the functional significance of co-operative DNA binding of the cytokine-driven transcription factor STAT1 (signal transducer and activator of transcription 1) in an experimental murine model of acute myocardial infarction (MI). STAT1 knock-in mice expressing a phenylalanine-to-alanine substitution at position 77 in the STAT1 amino-terminal domain were examined for the early clinical effects produced by ligation of the left anterior descending coronary artery (LAD), an established model for MI. The F77A mutation has been previously reported to disrupt amino-terminal interactions between adjacent STAT1 dimers resulting in impaired tetramerization and defective co-operative binding on DNA, while leaving other protein functions unaffected. Our results demonstrate that a loss of STAT1 tetramer stabilization improves survival of adult male mice and ameliorates left ventricular dysfunction in female mice, as determined echocardiographically by an increased ejection fraction and a reduced left intra-ventricular diameter. We found that the ratio of STAT3 to STAT1 protein level was higher in the infarcted tissue in knock-in mice as compared to wild-type (WT) mice, which was accompanied by an enhanced infiltration of immune cells in the infarcted area, as determined by histology. Additionally, RNA sequencing of the infarcted tissue 24 h after LAD ligation revealed an upregulation of inflammatory genes in the knock-in mice, as compared to their WT littermates. Concomitantly, genes involved in oxidative phosphorylation and other metabolic pathways showed a significantly more pronounced downregulation in the infarcted tissue from STAT1^F77A/F77A^ mice than in WT animals. Based on these results, we propose that dysfunctional STAT1 signalling owing to a lack of oligomerisation results in a compensatory increase in STAT3 expression and promotes early infiltration of immune cells in the infarcted area, which has beneficial effects on left ventricular remodelling in early MI following LAD ligation.

## Introduction

Acute myocardial infarction (MI) is considered a major cause of mortality and disability worldwide (1). The irreversible cell damage following myocardial injury results from an abrupt reduction in coronary blood flow, which exposes the cells to hypoxia, culminating in tissue necrosis and a severe inflammatory response (2, 3). Several molecular mediators contribute towards establishing this inflammatory microenvironment at the infarct site, including tumor necrosis factor (TNF), interferon gamma (IFNγ), interleukin 6 (IL-6) and other cytokines, which elicit their intracellular effects through transcription factors such as nuclear factor kappa B (NF-κB) and signal transducers and activators of transcription (STATs), respectively (4).

STATs are latent cytoplasmic transcription factors, activated by cytokines such as interferons and interleukins upon binding to their cognate receptor-kinase complexes (5, 6). These proteins have a modular domain structure divided into six domains, namely (1) an amino-terminal domain, (2) a coiled-coil domain, (3) a DNA-binding domain, (4) a linker domain, (5) an Src-homology 2 (SH2) domain, and (6) a carboxyl-terminal transactivation domain (7, 8). Ligand binding to cytokine receptors triggers transmembrane receptor dimerization and auto-phosphorylation of receptor-associated Janus kinases (JAKs). JAKs additionally phosphorylate the cytoplasmic tails of these receptors to serve as docking sites for STAT proteins, which are then phosphorylated at a critical tyrosine residue in their transactivation domain. The activated STATs subsequently translocate as phosphorylated dimers to the nucleus, where they bind as homo- or heterodimers to specific high-affinity, palindromic DNA sequences called IFN-gamma-activated sites (GAS) within the promoter regions of their target genes (9). Both phosphorylation and nuclear translocation of STATs are fast processes, occurring within minutes of cytokine stimulation. Active STATs are retained in the nucleus, only to return to the cytosol upon dephosphorylation by nuclear phosphatases, where they are rephosporylated at active receptors and participate in an additional round of nucleocytoplasmic translocation (10).

In the context of MI-triggered early inflammation, a large array of cytokines and growth factors produced by cells recruited to the infarct site are sensitive to IFN-activated JAK–STAT signalling (11, 12). Among the seven-member STAT family, studies report the expression of STAT1, STAT3, STAT5a and STAT5b in the heart (13, 14). Even though STAT proteins show a high sequence homology, they exhibit a substantial functional difference in mediating ischaemia/reperfusion injury in the myocardium (15, 16). STAT3 has been widely reported to exert a cardio-protective effect on cardiac pathogenesis during endotoxic shock and ischaemia through the induction of cardiac hypertrophy and anti-apoptotic genes like *Bcl-2* and *c-Fos*, which mediate cell survival (15, 17–19). On the contrary, STAT1 has been shown to mediate apoptosis in cardiomyocytes during ischaemia/reperfusion injury through activation of caspases-2, −9, and −3 (20, 21). Compared to WT counterparts, cardiomyocytes from STAT1 knock-out mice display increased autophagy in the peri-infarct region (22), and, in addition, STAT-1-deficient cells are resistant to TNF-induced apoptotic death (23).

Co-operative DNA binding of STAT1 is a hallmark in IFNγ (type II) signalling and requires the formation of stable tetramers on adjacent GAS sites on DNA (24). A substitution of phenylalanine to alanine at position 77 (F77A) in the amino-terminal domain of STAT1 has been shown to result in defective co-operative DNA binding due to a lack of tetramer formation (24–26). Mice with homozygous expression of the STAT1-F77A mutation displayed impaired STAT1 co-operativity resulting in a strongly reduced type II IFN-mediated anti-bacterial immunity. In contrast, anti-viral responses are essentially unaltered in this transgenic mouse line, since the type I IFNα signal pathway involves the formation of a STAT1/STAT2 heterodimer, also known as interferon-stimulated gene factor 3 (ISGF3), which binds specifically to non-palindromic ISRE (IFNα-stimulated response elements) sites without tetramer formation (25).

Given that, in cytokine-exposed cells, STAT1 transcriptionally upregulates its own expression *via* a positive feed-back loop, the protein levels of endogenous STAT1 may differ between the two genotypes. Based on these observations, we investigated the effects of a sterile inflammatory insult such as acute MI on tissue repair in the STAT1^F77A/F77A^ mouse line. In this study, we analyzed echocardiographic and transcriptomic profiles of co-operativity-deficient mice and their WT littermates following anterior descending coronary artery (LAD) ligation.

## Materials and methods

### Animals

In this study, we used the transgenic STAT1-F77A knock-in mouse line, which was generated as described in our previous publication (26). The STAT1 mutant mouse line does not exhibit any baseline pathology and has a normal life expectancy. All animal experiments were carried out in accordance with the German Animal Protection Act and approved by the local animal experimentation ethics committee (Niedersächsisches Landesamt für Verbraucherschutz und Lebensmittelsicherheit, 13/1226). Mice were housed at the animal facilities of the University Medical Centre Göttingen and kept on a 12-h day/night cycle with standard chow and water *ad libitum*. Littermates aged 8–12 weeks carrying a WT allele were obtained from STAT1-F77A heterozygous crossings. Three days before LAD surgery, metamizol (2 mg/mL) was added to the drinking water to provide basic analgesia. Mice were sacrificed through CO_2_ asphyxiation at the indicated time points for harvesting hearts.

### LAD ligation surgery

Myocardial infarction was induced by permanent ligation of the left anterior descending coronary artery (LAD). Mice were anaesthetized by intraperitoneal injection of 10 μL/g body weight of a narcotic solution (50 μg/mL medetomidine, 500 μg/mL midazolam, 5 μg/mL fentanyl), and stabilized in the supine position under artificial respiration (150 μL volume, 150 strokes/min). Thoracotomy was performed in the fourth left intercostal space. LAD ligation was done to induce infarction, using Ethilon 9–0 BV-4 5.0 mm 3/8c EH7448G surgical suture. Cardiac apex discoloration into white was checked as a measure of proper occlusion of the coronary vessel. Sham-operated mice underwent the same procedure but without LAD occlusion. Following successful ligation, retractors were disconnected and suturing of the skin was performed using Prolene 6-0 C-1 13 mm 3/8c 8889H surgical silk. Narcotic antagonist (250 μg/mL atipamezol hydrochloride, 50 μg/mL flumazenil, in sterile 0.9% NaCl) was injected i.p. at a dose of 10 μL/g body weight. Mice were given a buprenorphine solution (3.24 μg/mL buprenorphine hydrochloride in sterile 0.9% NaCl) at a dose of 15 μL/g body weight and left on a pre-warmed 37°C plate until fully awake.

### Echocardiographic assessment

Transthoracic echocardiography was performed ≤7 days before LAD surgery and at 3 days, 1 week and 4 weeks after surgery. Mice were treated with 1.5% isoflurane anaesthesia, and then subjected to echocardiography in a supine position. Measurements were done using the Vevo x2100 system with a 30 MHz centre frequency ultrasound transducer (Visualsonics, Toronto, Canada). Core temperature was maintained at 37°C and heart rate was kept consistent between experimental groups (320–450 bpm). The long axis was visualized in B mode only, while the short axis was visualized in B and M mode. Data analysis was performed using the Vevo x2100 software. In the long axis, the diastolic (subscript d) and systolic (subscript s) lengths were determined (L_d_ and L_s_). In the short axis, left ventricular inner diameters and areas (LVID_s_, LVID_d_, Area_s_, and Area_d_) as well as anterior and posterior wall thickness were measured in systole and diastole. From these measurements, left ventricular functional parameters were calculated as follows: fractional area shortening (FAS) = 100 × (Area_d_–Area_s_) ÷ Area_d_, fractional shortening (FS) = 100 × (LVID_d_–LVID_s_) ÷ LVID_d_, end-systolic volume (Vol_s_) = (5/6) × (Area_s_ × L_s_), end-diastolic volume (Vol_d_) = (5/6) × (Area_d_ × L_d_), and ejection fraction (EF) = 100 × (Vol_d_ –Vol_s_) ÷ Vol_d_.

### Protein extraction and Western blotting

Hearts were explanted at the indicated time points after LAD surgery. The main infarcted area in the lower third of the left ventricle was separated, and rapidly frozen in liquid nitrogen. Roughly 40 mg of tissue were lysed in 800 μL radioimmunoprecipitation assay buffer (RIPA) buffer, supplemented with 1% complete protease inhibitor cocktail (Merck/Millipore), 1% phenylmethylsulfonyl fluoride (PMSF) (100 μM in isopropanol), and 1 mM Na_3_VO_4_. Lysates were then homogenized using the Speedmill tissue homogenizer (Analytik Jena, Jena, Germany) with innuSpeed lysis tubes A for 4 × 45 s. The homogenized samples were incubated on ice for 30 min, and then transferred to a fresh reaction tube before sonicating thrice at 50% amplitude for 30 s. After sonication, debris was spun down at 12,000 g for 20 min at 4°C. Supernatants were collected as protein extracts and stored at −80°C until used. Forty μg of each sample was boiled for 5 min at 95°C in 6 × Laemmli buffer, before being loaded onto a 10% sodium dodecyl sulfate-polyacrylamide gel (SDS-PAGE), and subsequently transferred to a polyvinylidene fluoride (PVDF) membrane. The membranes were incubated with a primary antibody, followed by incubation with a conjugated secondary antibody (LI-COR). Bound immunoreactivity was detected with anti-rabbit IRDye 800CW antibodies and visualized on a LI-COR Odyssey CLx imaging machine. The following primary antibodies were used: anti-GAPDH (1:5,000, Cell Signaling Technology, #2118), anti-STAT1 (1:1,000, Cell Signaling Technology, #14994), anti-phosphotyrosine-STAT1 (1:1,000, Cell Signaling Technology, #9167), anti-STAT3 (1:750, Cell Signaling Technology, #30835), and anti-phosphotyrosine-STAT3 (anti-p-STAT3, 1:1,000, Cell Signaling Technology, #9145). For each sample, STAT levels were normalized to the band intensity of the GAPDH band, and quantifications were made by normalizing protein levels to sham-treated WT mice, which were used as reference.

### Immunohistochemistry and histology

Freshly isolated hearts were fixed in 4% formaldehyde solution, then dehydrated, embedded in paraffin and sectioned. Serial sections of 5 μm thickness were stained after deparaffinization and rehydration, using rabbit monoclonal antibodies raised against phosphotyrosine-STAT1 (1:200, Cell Signaling Technology, #9167), STAT1 (1:200, Cell Signaling Technology, #14994), STAT3 (1:200, Santa Cruz Technology, sc-7179), myeloperoxidase (MPO, 1:200, Thermo Fisher, PA5-16672), CD3 (1:200, Bio-Rad, MCA1477) and CD68 (1:75, Bio-Rad, MCA1957T). Primary antibodies were diluted in 10% foetal bovine serum (FBS) in phosphate-buffered saline (PBS) and incubated over night at 4°C, while biotinylated anti-rabbit or anti-rat secondary antibody (DAKO, Glostrup, Denmark) diluted at 1:1,000 in 10% FBS in PBS were incubated for 1 h at room temperature. Detection of bound immunoglobulins was achieved with the ABC method employing the biotinylated secondary antibodies and avidin-horseradish peroxidase complexes (Vectastain Elite ABC HRP). Diaminobenzidine (DAB) was used as a substrate for visualizing the enzymatic reaction that produces a brown precipitate. Finally, the sections were counterstained with Mayer’s haematoxylin.

Serial sections were taken roughly 1.2, 1.6, 2.0, and 2.4 mm from the apex of the heart, corresponding to the entire region of the infarcted area. For H&E staining, the slides were exposed to a filtered Mayer’s haematoxylin solution and subsequently counterstained in 0.1% eosin solution. The samples were mounted using Entellan (Merck Chemicals , Darmstadt, Germany) and analyzed using light microscopy. The distribution of STAT staining in heart tissues was assessed in a semi-quantitative manner using the following criteria: 0; no specific staining, 1; small and restricted areas of inflammation of less than half the left ventricular wall, 2; distribution of inflammatory foci up to 90% of the left ventricular wall, 3; entire left ventricle with little or no involvement of septum and right ventricle, and 4; entire left ventricle and strong infiltration of the right ventricle or the septum. Assessment of STAT staining intensity was as follows: 0: no specific staining, 1: single cells at low density, 2: moderate cell density, 3: high cell density with numerous positively stained cells, and 4: highest cell density of positively stained cells (>80%). Using an arc measure, the size of the myocardial infarct was determined as the segment of a circle around the circumference of the heart that completely encloses the cellular infiltrate.

### Fluorescence-activated cell sorting

For FACS analysis, cells of spleen and lymph nodes were stained after preparing a single cell suspension with 5 × 10^5^ cells used for analysis. The cells were centrifuged at 185 g for 8 min and 4°C and subsequently washed once with FACS buffer (2% BSA, 308 mM NaN_3_ in PBS). They were stained by resuspension in 100 μL antibody mastermix (FACS buffer with antibodies) for 15 min at 4°C, while being protected from light. Cells were washed again using FACS buffer and were resuspended in 1 mL FACS buffer before analysis. For identifying T regulatory cells, staining of nuclear forkhead box P3 (FoxP3) was performed using the APC-conjugated anti-mouse/rat/human FOXP3 Flow kit (clone FJK 16 s, BioLegend) according to the manufacturer’s recommendations. Cells were co-stained with anti-CD25-FITC (clone 7D4, BD Pharmingen, Franklin Lakes, NJ, United States) and anti-CD4-PE/Cy5 (clone H129.19, BioLegend, San Diego, CA, United States) and incubated for 30 min with 400 μl Fix-Perm solution (Becton Dickinson), while again protected from light throughout the entire staining. Cells were washed once in FACS buffer. Blocking was performed for 15 min in 2% rat serum in Perm buffer (Becton Dickinson). One μL of FoxP3 antibody was then directly added and incubated with the cells for 30 min. Cells were washed twice in Perm buffer and resuspended in 200 μL FACS buffer. Sorting was performed using the FACSCalibur cell sorter (Becton Dickinson). For analysis, the software CellQuestPro and FlowJo Version 10.8.0 (Becton Dickinson) were used. A minimum of 30,000 cells was sorted for quantitative analysis (Supplementary Figure 1). A live/dead stain was not included in the experiments. Regulatory T cells were quantified using CD25 FITC (1:200), CD4 PE/Cy5 (1:500) and the PE anti-mouse/rat/human FOXP3 Flow kit (BioLegend, San Diego, CA, United States).

### RNA-seq transcriptome analysis

Whole hearts from female mice were extracted 24 h after LAD surgery, and the LV infarcted area was excised with a surgical blade. Samples (*n* = 12) were collected from four groups comprising of three animals from each genotype (WT versus F77A) and treatment type (MI versus sham). Using these replicates allowed for the estimation of within sample variability of gene expression to make inferences between samples from both genotypes and treatment types. Total RNA was extracted in TRIzol reagent (Thermo Fisher Scientific), before they were stored at −80°C until further analysis. RNA quantity and quality were assessed with the Fragment Analyzer (Advanced Analytical Technology), using the Standard Sensitivity RNA analysis kit (DNF-471, Agilent Technologies). The yield of the extracted RNA from all samples ranged from 15.8 ± 3.0 to 45.5 ± 6.5 μg. RNA-seq libraries were prepared with 500 ng of total RNA, using an Illumina TruSeq stranded mRNA preparation kit. All samples selected for sequencing exhibited an RNA integrity number over 8 (with a range from 8.03 to 10.0). A modified strand-specific massively parallel cDNA sequencing mRNA-seq protocol was followed. Libraries were quantified using the QuantiFluor dsDNA system (Promega) and were pooled and sequenced on an Illumina HiSeq 4000 generating 50 bp single-end reads (30–40 million reads/sample). Reads were aligned for each sample to the mouse reference genome (GRCm38, mus_musculus_mm10) using STAR software, allowing for a maximum of 2 mismatches per 50 bp read. Read counts per gene were obtained using featureCounts (version 1.4.5-p1). Differential expression analysis was performed using the Benjamini-Hochberg procedure implemented in the R/Bioconductor package DESeq2 (version 3.4.2) (27). Differentially expressed (DE) genes had an absolute log_2_(fold-change) >1 and a false discovery rate (FDR)-adjusted value of *p* < 0.05. Gene annotation was done using biomaRt R package (28). For gene set enrichment analysis (GSEA) and data visualization, ggplot2, kableExtra, pheatmap, gage, dplyr, pathview, biomaRt, grid, VennDiagram, and ggrepel R packages were used. The raw RNA-Seq data are available from the corresponding author.

### Statistical analysis

For statistical analysis, IBM SPSS (Chicago, IL, United States), SigmaPlot (Systat Software, Erkrath, Germany), and R studio were used. The data are expressed as means ± standard error. The statistical analysis was performed by a two-tailed *t*-test or one-way ANOVA with Tukey’s post-hoc analysis for multiple comparisons. Survival analysis was performed using the Kaplan-Meier method, and between-group survival differences were tested using the log-rank (Mantel-Cox) test. A value of *p* of ≤0.05 was used to indicate statistical significance.

## Results

### STAT1^F77A/F77A^ mice are protected from adverse cardiac remodelling

**Figure 1:**
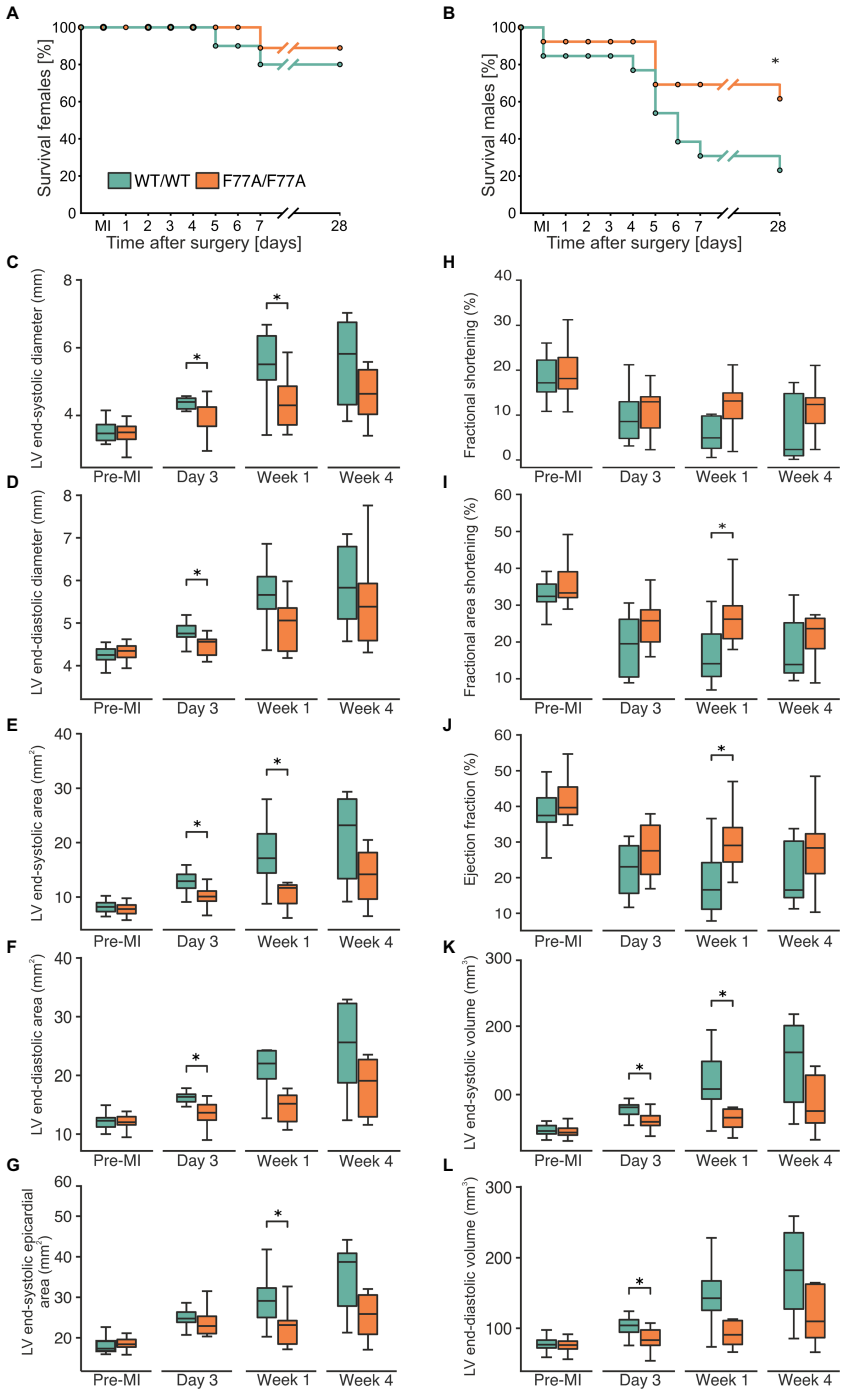
The STAT1-F77A mutation protects mice from adverse remodelling after myocardial infarction. **(A,B)** Kaplan-Meier curves showing the survival rates of female **(A)** and male **(B)** mice expressing homozygously either STAT1 WT (green) or the amino-terminal F77A mutation (orange) after experimental FIGURE 1 (Continued)MI due to LAD ligation. Log-rank test was used to test for a difference in survival between the two genotypes. **(C–L)** Echocardiographic assessment of haemodynamic parameters in WT (green) and STAT1^F77A/F77A^ (orange) female mice before LAD ligation (*n* = 23 versus *n* = 22), 3 days (*n* = 12 versus *n* = 11), 1 week (*n* = 9 versus *n* = 8) and 4 weeks (*n* = 7 versus *n* = 8) following surgery. The box plots demonstrate data distribution between the two genotypes in left ventricular **(C)** end-systolic diameter, **(D)** end-diastolic diameter, **(E)** end-systolic area, **(F)** end-diastolic area, **(G)** end-systolic epicardial area, **(H)** fractional shortening, **(I)** fractional area shortening, **(J)** ejection fraction, **(K)** end-systolic volume, and **(L)** end-diastolic volume. Asterisks indicate statistically significant differences at a *p*-level of ≤0.05, as assessed by a two-tailed Student’s *t*-test.

Cardiac remodelling following MI involves structural and functional changes, including hypertrophy of the non-affected, viable myocardium, accompanied by LV dilatation and reduced ejection fraction. To study the role of co-operative DNA binding of STAT1 in left ventricular remodelling, we induced MI in mice carrying the amino-terminal F77A mutation as well as in their WT littermates by LAD ligation surgery. Kaplan-Meier analysis demonstrated similarly high post-MI survival rates in female mice irrespective of the genotype (Figure 1A). However, as compared to their WT counterparts, the survival rate of male mice carrying a homozygous F77A mutation was significantly higher (Figure 1B). In addition, we confirmed a sex-specific difference in survival following LAD operation already known from the literature, wherein WT female mice demonstrated a better 4-week survival rate when compared to male mice (29, 30). Given the increased survival rate of F77A as compared to WT male mice, we performed subsequent experiments on female mice, which ensured optimal sample sizes and avoided a selection bias.

Using echocardiography, we observed that before LAD ligation the haemodynamic parameters did not significantly differ between the two genotypes. Given the known sex-specific difference in survival rates, with male WT mice having the highest mortality, we exclusively performed transthoracic echocardiography in operated female animals before and at day 3, week 1, and week 4 after LAD ligation to monitor cardiac function in both genotypes, compared with pre-operative measurements used as reference. Data showed that left ventricular end-systolic and end-diastolic inner diameters (Figures 1C,D) as well as cross-sectional areas (Figures 1E–G) were all significantly lower in STAT1^F77A/F77A^ female mice when compared to WT counterparts at either day 3 and/or week 1 (Supplementary Table 1). This cardiac improvement in the knock-in mice continued as a non-significant trend at week 4 (Figures 1C–G). Accordingly, in comparison to WT mice, the fractional area shortening and the ejection fraction were significantly less impaired in STAT1^F77A/F77A^ female mice 1 week after LAD ligation (Figures 1H–J). Furthermore, the left ventricular end-systolic volume was significantly reduced in the knock-in mice, as was the end-diastolic volume at day 3 after LAD ligation (Figures 1K,L). From these observations, we conclude that a defect in STAT1 co-operative DNA-binding substantially ameliorated cardiac remodelling in MI-treated female mice, resulting in a better haemodynamic outcome.

### Knock-in mice show a higher STAT3/STAT1 ratio in the infarcted myocardium

**Figure 2:**
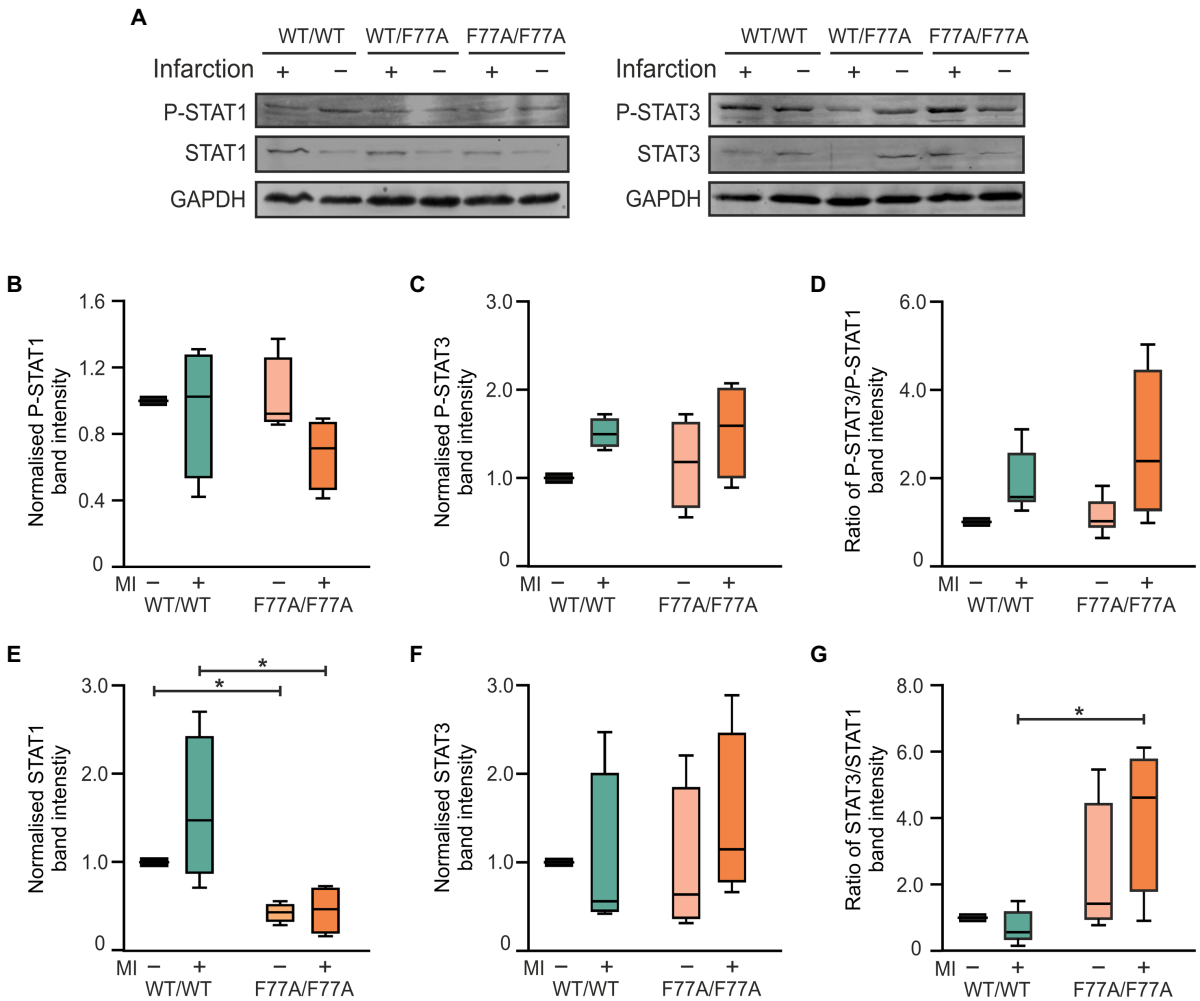
Increased ratio of total STAT3 to STAT1 level in the myocardium of mice expressing homozygously the STAT1-F77A mutation on day 3 after MI. **(A)** Representative Western blots of proteins extracted from either infarcted ventricular tissue (+) or unaffected areas (−) from the ventricles of mice carrying the WT/WT, WT/F77A and F77A/F77A genotypes. Blots were probed with anti-phosphotyrosine STAT1 (p-STAT1), anti-pan STAT1 (STAT1), anti-phospho-STAT3 (p-STAT3) and anti-pan STAT3 antibodies, as well as a GAPDH-specific antibody (GAPDH) used for normalization (see also Supplementary Figure 3). **(B**–**G)** Quantification of immunoblots from three independent experiments, as shown in **(A)** and standardized to the WT non-infarcted value. Only the α-forms of STAT1 and STAT3 were evaluated. Asterisks in the box plots indicate statistically significant differences, as determined using *t-*test. All WT bands were normalized to 1.

Given the echocardiographic results of an improved cardiac function in the MI-treated knock-in mice, we investigated the beneficial effects of the F77A mutation on tissue remodelling at a molecular level. For this purpose, snap-frozen infarcted areas of the hearts were collected at day 3 post-MI to evaluate cardiac inflammation during the acute phase of MI using biochemical analyses. Protein lysates of the infarcted areas were compared with extracts isolated from unaffected basal parts of the ventricles by means of Western blotting using phospho-STAT1, phospho-STAT3, and their corresponding pan-STAT1 and pan-STAT3 specific antibodies (Figure 2A). On day 3 post-MI, we observed similar tyrosine phosphorylation levels of STAT1 and STAT3 in the infarcted heart tissues from WT and STAT1^F77A/F77A^ mice (Figures 2B–D). As compared to the WT mice, we measured a significantly lower expression of total STAT1 in the infarcted myocardial tissue of mice carrying the STAT1^F77A/F77A^ alleles (Figure 2E). There was a non-significant change in the level of STAT3 (Figure 2F) and a significantly higher ratio of total STAT3 to STAT1 level in the ischaemic myocardium of STAT1^F77A/F77A^ mice (Figure 2G). Based on these data, we hypothesized that a higher ratio of STAT3 to STAT1 level possibly drives the improved survival and cardiac function in STAT1^F77A/F77A^ mice following MI.

### STAT1^F77A/F77A^ mice exhibit a pronounced immune cell infiltration in the ischaemic heart

**Figure 3:**
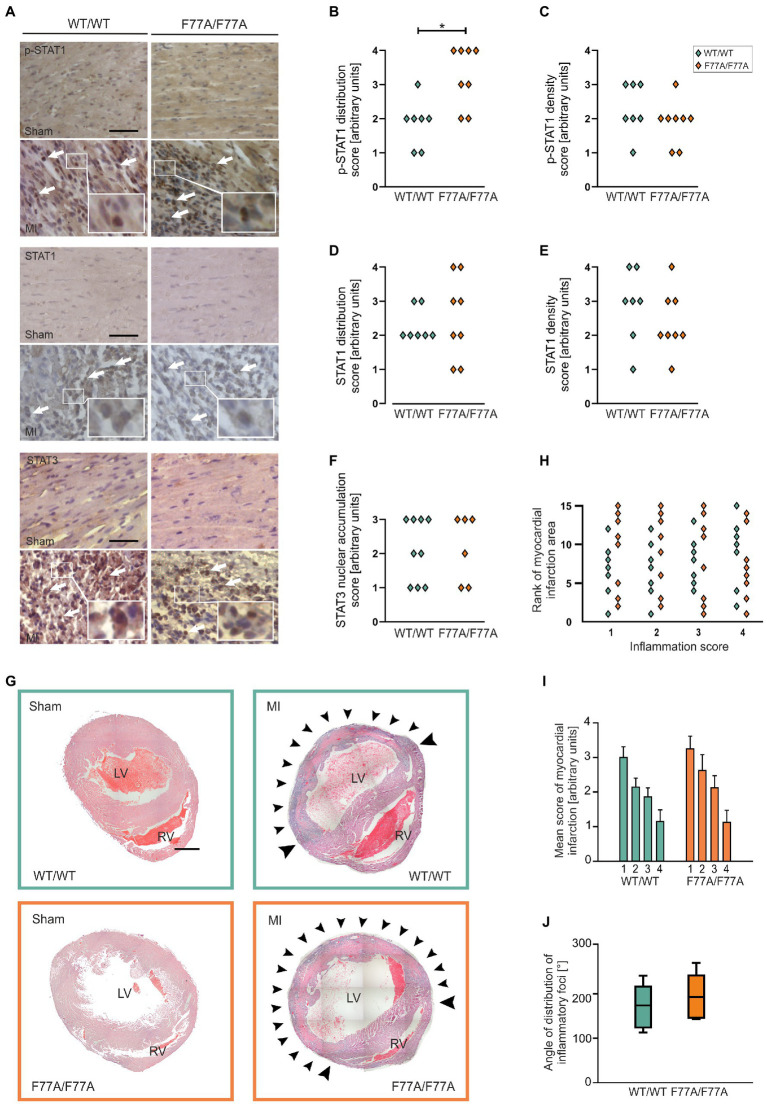
A pronounced accumulation of infiltrating phospho-STAT1-expressing cells in the myocardium after MI, as assessed by means of histological analysis. **(A**–**G)** Immunohistochemical staining of p-STAT1, STAT1 and STAT3 in heart sections on day 3 after MI or sham operation. **(A)** Representative micrographs of the left ventricular wall of sham-(top panel) and MI-operated animals (bottom panel) in mice carrying the WT/WT and F77A/F77A genotype, respectively (scale bar: 50 μm). Arrows mark individual cells with positive immunohistochemical staining for the indicated antigen FIGURE 3 (Continued)(WT/WT male *n* = 4, female *n* = 3; F77A/F77A male *n* = 5, female *n* = 3). **(B**–**F)** Quantifications of the distribution of positively stained cells over the ventricular wall and septum (left panel), and of the density of positively stained cells within the infarcted area (right panel). Asterisks indicate statistically significant differences, as determined using *t-*test. **(G**–**J)** Haematoxylin and eosin (H&E) staining of paraffin-embedded cardiac tissue sections at day 3 after MI or sham operation. **(G)** Representative micrographs of hearts demonstrating the infiltration of immune cells in the infarct area. Strong infiltration of the LV myocardium is visible in the two genotypes after infarction, but not in the hearts of sham-operated animals. Arrowheads mark representative areas of infiltration. LV, left ventricle; RV, right ventricle. Images are composites of 15–20 individual images with arrowheads pointing to the infiltrate (scale bar: 1 mm, *n* = 8 for each genotype). **(H)** Scatter blot of the ranks assigned to each sample with experimental myocardial infarction in serial sections near the apex (1, ~1.2 mm from apex), in the middle of the infarcted area (2, ~1.6 mm from the apex), in the upper part of the infarcted area (3, ~2 mm from the apex) and in the uppermost part of the infarcted area (4, ~2.4 mm from the apex). Each point corresponds to one heart at the four anatomical heights. The largest infarction was assigned the highest score. **(I**,**J)** Evaluation of the extent of inflammation from H&E staining of paraffin-embedded cardiac sections, 3 days after MI. **(I)** An arbitrary score was assigned to each infarcted heart in serial sections, near the apex (1, ~1.2 mm from apex), middle of the infarct zone (2, ~1.6 mm from the apex), upper part of the infarct zone (3, ~2 mm from the apex) and above the infarct zone (4, ~2.4 mm from the apex). The scores assigned were “0” (no inflammation), “1” (small, restricted areas of inflammation in less than half of the LV wall), “2” (distribution of inflammatory foci up to 90% of the LV wall), “3” (entire LV inflamed with little or no involvement of septum or RV) and “4” (inflamed LV including parts of the septum and RV). Error bars represent standard error of the mean. **(J)** The distribution of inflammatory infiltrates was assessed by determining the angle from the LV centre, where pronounced accumulation of immune cells was located. The angle was determined for sections in the middle of the infarcted area. Box plots denote the median value with a line, while boxes contain the 25th and 75th percentiles and the whiskers mark the interquartile range from the 5th to 95th percentile (*n* = 7 for WT and *n* = 8 for F77A/F77A knock-in groups).

After the assessment of STAT1 and STAT3 levels and tyrosine phosphorylation using Western blotting, we further investigated the impact of impaired STAT1 co-operative DNA-binding on acute MI using immunohistochemical staining. Paraffin-embedded hearts of WT and STAT1^F77A/F77A^ mice obtained 3 days after LAD ligation or sham-operated mice were sectioned and stained for phospho-STAT1, total STAT1, and total STAT3. Analysis of staining intensity and distribution revealed high numbers of both STAT1- and STAT3-positive cells, which were widely distributed across the infiltration in the infarcted area, characteristic of high-grade inflammation (Figure 3A). Negative staining of STAT1 and STAT3 was observed in normal myocardial tissue outside the ischaemic area in MI-treated mice and in sham-operated mice, confirming a pathophysiological link between inflammatory responses at the infarct site and the activation of JAK–STAT signalling in the affected heart. Interestingly, we observed an increased distribution of tyrosine-phosphorylated STAT1-positive immune cells in the MI-treated STAT1^F77A/F77A^ mice over a wider area as compared to their STAT1^WT/WT^ littermates (Figure 3B). However, the densities and distribution of STAT1-expressing cells did not differ between the two genotypes (Figures 3C–E), as was the extent of STAT3 nuclear accumulation (Figure 3F).

To investigate the size of the infarcted area 3 days after LAD ligation, we examined the extent of immune infiltration over the circumference of the affected left ventricle. Haematoxylin-eosin staining of total heart sections from MI-treated mice at day 3 after LAD ligation showed a pronounced myocardial accumulation of infiltrating immune cells in the affected myocardium in all genotypes, which was absent in sham-operated animals (Figures 3G,H). The STAT1^F77A/F77A^ group was non-significantly higher in a consecutive ranking for the extent of inflammation as compared to the WT group (Figure 3H), confirming that STAT1^F77A/F77A^ mice had a slightly, albeit non-significantly larger extent of inflammation (Figure 3I). Similarly, the angle of the circumference affected by the inflammatory infiltrate was broader in the transgenic mice, although again this did not reach statistical significance (Figure 3J). There was no fibrotic tissue formation at day 3 after LAD ligation, as judged from the absence of scar formation using Masson trichrome staining (data not shown). Thus, we conclude that WT and STAT1^F77A/F77A^ genotypes had both similar infarct sizes during the acute phase of inflammation at day 3 after LAD ligation.

**Figure 4:**
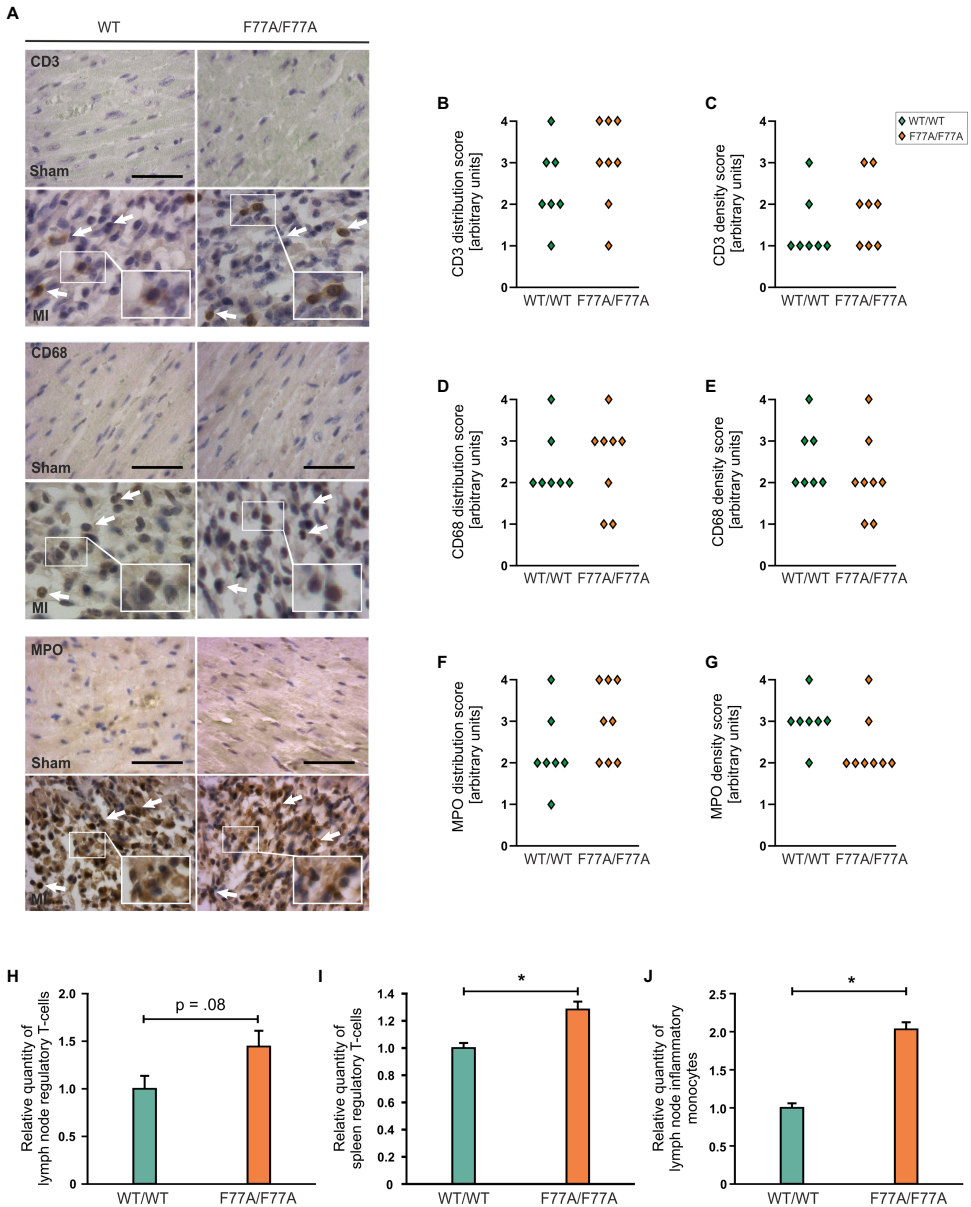
Recruitment of different immune cells in WT and STAT1^F77A/F77A^ mice. **(A)** Immunohistochemical staining of immune cell subsets in STAT1^F77A/F77A^ mice with antibodies against CD3 (upper panel), CD68 (middle panel), and myeloperoxidase (MPO) (lower panel) as markers for T cells, monocytes and neutrophil granulocytes, respectively, on day 3 following MI or sham operation. Representative LV wall micrographs of sham-operated animals (top) and mice subjected to LAD ligation (bottom) from the two genotypes (scale bar: 50 μm, WT/WT male *n* = 4, female *n* = 3; F77A/F77A male *n* = 5, female *n* = 3). Arrows mark positively stained cells expressing the indicated antigen. Arbitrary quantification of the **(B)** distribution of CD3-positive cells and their **(C)** density over the ventricular wall and septum. Quantification of the distribution **(D**,**F)** and density **(E**,**G)** of CD68-positive **(D**,**E)** and MPO-positive cells **(F**,**G)** (WT/WT male *n* = 4, female *n* = 3; F77A/F77A male *n* = 5, female *n* = 3). **(H–J)** Analysis of the relative numbers of regulatory T cells **(H**,**I)** and inflammatory monocytes **(J)** in lymph nodes **(H**,**J)** and spleen **(I)** of 9-week-old females from the STAT1^F77A/F77A^ mouse line as compared to WT controls. Asterisks indicate statistically significant differences (value of *p* ≤ 0.05; *t-*test).

Furthermore, we attempted to characterize different subsets of inflammatory cells during the acute phase of MI, by means of immunohistochemistry using horseradish peroxidase staining. To this end, tissue sections with inflammatory infiltrates were stained using antibodies against CD3 for infiltrating T cells, CD68 for monocytes and macrophages, and MPO to identify neutrophils. Immunocytochemistry from the infarct area showed that the population size of different immune cell subsets was not different between WT and STAT1^F77A/F77A^ mice (Figures 4A–G).

### Increased numbers of T regulatory cells in STAT1^F77A/F77A^ mice

In order to study the role of STAT1 DNA-binding co-operativity in regulating immune cell populations post-MI, we next analyzed the spleen and mediastinal lymph nodes of knock-in and WT female (9-week-old) mice for baseline populations of neutrophils, monocytes, T cells, and B cells using FACS. Flow cytometry analysis demonstrated that regulatory T cells in the lymph nodes and spleen of STAT1^F77A/F77A^ mice were mildly increased (Figures 4H,I). Furthermore, we found monocytes as a prevalent cell-type in the lymph nodes at day 3 (Figure 4J). In summary, regulatory T cells were significantly increased in the spleen and there was a trend towards a higher number of regulatory T cells in lymph nodes of the transgenic mouse line. In addition, monocytes were also increased in the lymph nodes of knock-in mice as compared to their WT littermates.

### Differential gene expression in the infarcted area of STAT1^F77A/F77A^ mice

**Figure 5:**
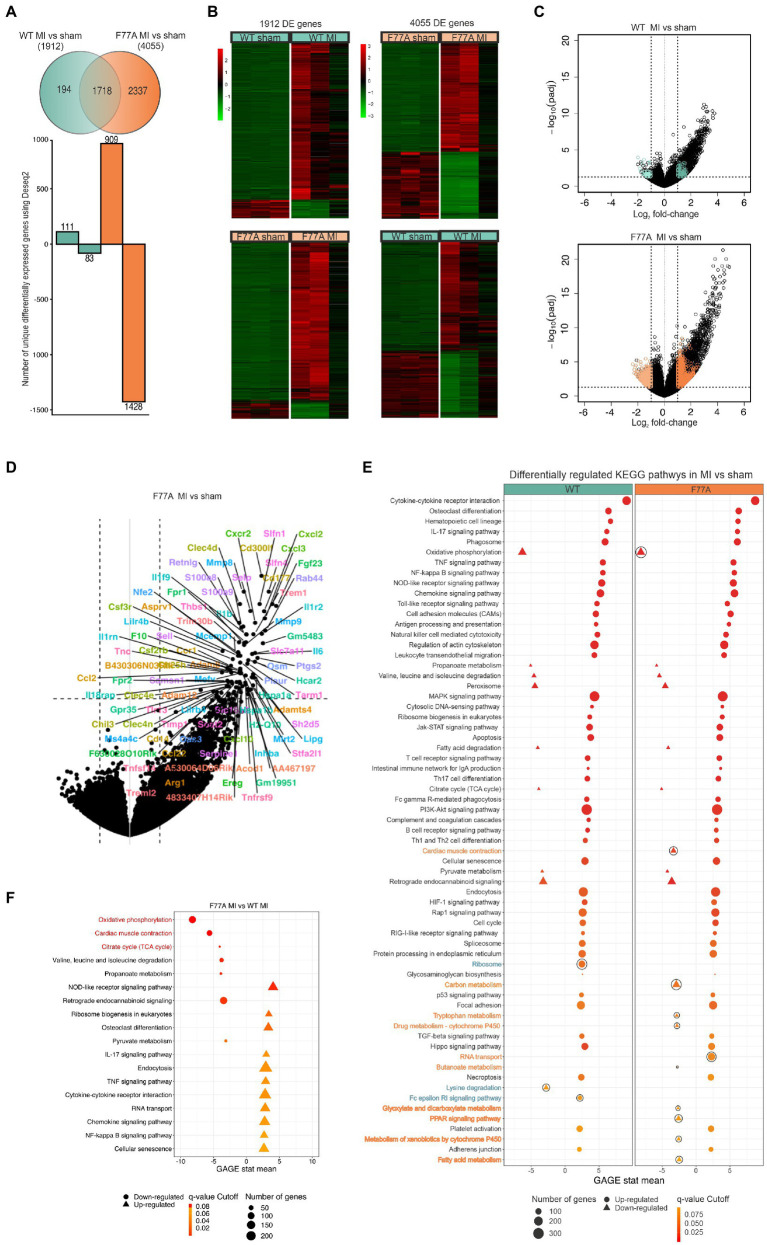
Higher number of differentially regulated genes in STAT1^F77A/F77A^ mice following LAD ligation compared to WT mice. **(A)** Venn diagram showing the number of unique genes that are upregulated or downregulated in WT and transgenic mice (*n* = 12, for each group 3 mice) 24 h after LAD ligation. FIGURE 5 (Continued)A log_2_(fold change) >1 and a value of *p* < 0.05 were used. Green shows all the differentially expressed genes in WT and orange in STAT1^F77A/F77A^ mice. **(B)** Hierarchical clustering of RPKM (reads per kilobase of transcript per million reads mapped) values in the WT and STAT1^F77A/F77A^ mice in both sham- and LAD-operated mice. First heat map was generated using a list of genes that are differentially expressed in WT (MI vs. sham) comparison (*n* = 1,912 genes). Second heat map is generated using a list of genes that are differentially expressed in STAT1^F77A/F77A^ (MI vs. sham) comparison (*n* = 4,055 genes). The R pheatmap package was used for visualization. **(C)** Volcano plot representation of differentially expressed genes in WT MI vs. sham-operated (top) and STAT1^F77A/F77A^ MI vs. sham-operated (bottom) mice. Every point represents an individual transcript in each plot. Colored points correspond to the uniquely differentially expressed genes in the following groups: 111 genes in WT MI vs. sham upregulated (dark green), 83 genes in wild-type MI vs. sham downregulated (light green), 909 genes in STAT1^F77A/F77A^ MI vs. sham upregulated (dark orange), 1,428 genes in F77A MI vs. sham downregulated (light orange). Padj: Benjamini-Hochberg adjusted value of *p*. **(D)** Identification of highly upregulated gene transcripts in the infarcted area of MI-operated STAT1^F77A/F77A^ mice using the gene expression signature (right). The plot is identical to (**C**, right) with each point indicating a gene-of-interest that displays both, a large-magnitude fold-change (x-axis) as well as high statistical significance (y-axis). **(E)** KEGG pathways regulated in response to LAD ligation in WT and STAT1^F77A/F77A^ mice. Pathways were retrieved by performing gene set enrichment analysis (GSEA) using the R package gage and visualized using the R package PathView. GSEA-enriched pathways that are significantly upregulated (*q*-value < 0.1) are displayed with circles, while downregulated pathways are shown as triangles. Outlined pathways represent unique KEGG pathways in the respective comparison STAT1^F77A/F77A^ MI vs. sham (dark orange) and WT MI vs. sham (dark green), respectively. **(F)** A list of KEGG differentially regulated pathways comparing MI-treated STAT1^F77A/F77A^ versus WT mice (*n* = 12) after implementing the gage package for gene set enrichment. Oxidative phosphorylation, cardiac muscle contraction, and citrate cycle are the top three downregulated KEGG pathways in the infarcted area of STAT1^F77A/F77A^ mice as compared to WT mice. A list of KEGG differentially regulated pathways comparing STAT1^F77A/F77A^ MI versus WT MI mice (*n* = 12) after implementing the gage package for gene set enrichment. GSEA-enriched pathways with *q*-value <0.1 are shown with top three highlighted in red. Circles represent downregulated pathways, while triangles represent upregulated ones.

Based on these findings, we hypothesized that STAT1 drives a pro-inflammatory program during myocardial infarction and that loss of STAT1 co-operativity may alter the transcriptional profile toward a cardioprotective pro-survival program during the acute phase of MI within 24 h after surgery. To assess the physiological role of dysfunctional STAT1 signalling on global transcriptional regulation during the early phase of MI and immune infiltration, we constructed 12 RNA libraries from infarcted tissues collected one day after LAD surgery in the two groups of WT and knock-in mice, respectively. Samples from WT and STAT1^F77A/F77A^ mice that underwent sham or LAD operation were pooled separately. The transcriptome analysis from infarcted tissues of knock-in and WT mice after LAD ligation revealed 4,055 differentially expressed genes (DEGs) in the STAT1^F77A/F77A^ group compared to 1,912 DEGs found in the WT group [log_2_(fold change) >1, value of *p* < 0.05] (Figure 5A). We further identified the top 20 genes that were differentially expressed in WT and STAT1^F77A/F77A^ animals with LAD ligation versus sham operation, and found a considerable overlap between the two genotypes, including heat shock proteins, chemokines and cytokines such as IL6 and their receptors (Supplementary Table 2). Differential expression (DE) analysis revealed 2,337 DEGs unique in the STAT1^F77A/F77A^ group versus only 194 DEGs that were unique in the WT group post-MI. Following LAD operation, 909 DEGs were upregulated in the STAT1^F77A/F77A^ group compared to 111 DEGs in the WT group. Furthermore, 1,428 DEGs were downregulated in the STAT1^F77A/F77A^ group compared to 83 DEGs in the WT group after LAD ligation, while 1,718 DEGs were common between WT and STAT1^F77A/F77A^ groups. Using hierarchical clustering analysis of RPKM (reads per kilobase of transcript per million mapped reads), we revealed distinctive gene clusters and subclusters of upregulated and downregulated DEGs in the four groups (Figure 5B). Volcano plots demonstrated that a higher number of MI-related genes were differentially regulated in STAT1^F77A/F77A^ mice as compared to WT (Figure 5C). Furthermore, upregulated genes in STAT1^F77A/F77A^ mice after MI belonged to cytokine regulatory pathways, as shown in Figure 5D.

To explore the functions of these identified DEGs, we ran gene ontology (GO) and gene-set enrichment analysis (GSEA) to test for coordinated differential expression over gene sets. Interestingly, we found PI3K-Akt and JAK–STAT signalling pathways among the top enriched signal transduction pathways in both genotypes following MI (Figure 5E). All differentially expressed genes in STAT1^F77A/F77A^ animals from the MI vs. sham comparison were grouped into four main categories, namely immune processes, signal transduction, cellular processes, and metabolism (Supplementary Table 3). The top enriched immune processes, that were upregulated post-MI in both knock-in and WT mice, were cytokine-cytokine receptor interactions, chemokine signalling pathways, cell adhesion molecules, regulation of actin cytoskeleton, and leukocyte transendothelial migration (Figure 5E; Supplementary Table 4).

### Infarcted myocardium from STAT1^F77A/F77A^ mice reveals an immunologically active microenvironment

**Figure 6:**
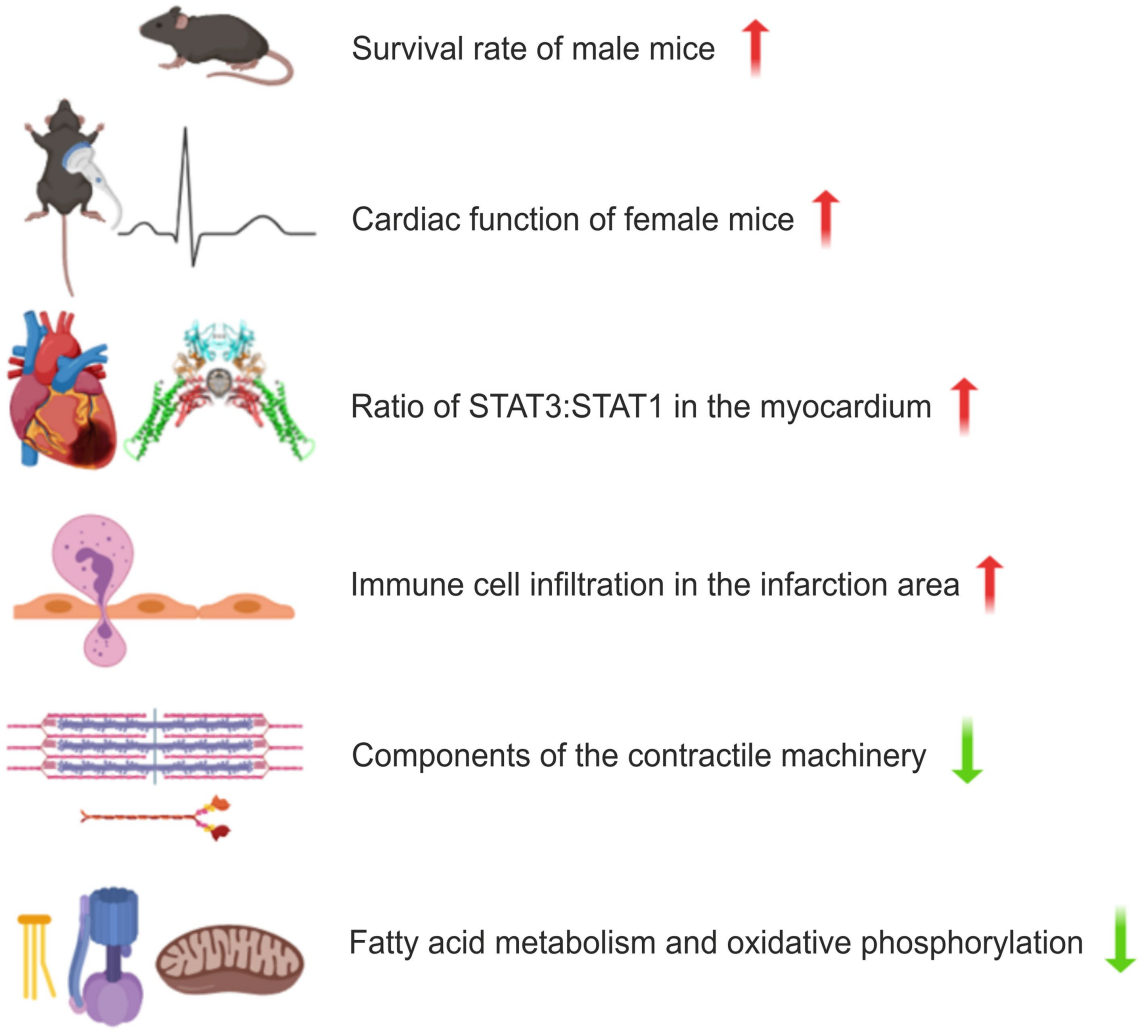
Schematic presentation of the effects of disrupted co-operative DNA binding in a murine model of myocardial infarction (MI). Male mice with a homozygous F77A mutation show a higher survival rate following left anterior descending artery (LAD) ligation, while the haemodynamic function of the ischaemic heart is less affected in female mice. Changes in the STAT protein levels as well as differentially regulated KEGG cellular processes are depicted for female STAT1^F77A/F77A^ mice. Upregulated pathways are displayed in red and downregulated pathways are in green. In the comparison between the transcriptomes from ischaemic and non-ischaemic myocardial tissues, prominent genotype-specific differences were observed for cardiac muscle contraction, fatty acid metabolism and oxidative phosphorylation.

Next, we compared expression patterns in infarcted areas across genotypes and found that oxidative phosphorylation, cardiac muscle contraction, and citrate cycle were the top three downregulated Kyoto Encyclopedia of Genes and Genomes (KEGG) pathways in STAT1^F77A/F77A^ mice, when compared to WT littermates (Figure 5F). The massive infiltration of neutrophils and other immune cell subsets in the infarcted myocardium possibly drives the downregulation of the cardiac muscle contraction pathway in STAT1^F77A/F77A^ mice. The analysis of DEGs across genotypes using a higher cut-off criteria identified a list of immune markers that were differentially regulated in STAT1^F77A/F77A^ mice. These immune markers clustered into groups, such as immune-activating chemokines and their receptors (e.g., *Ccl2*, *Ccl22*, *Ccr1*, *Cxcl10*, *Cxcl2*, *Cxcl3*, and *Cxcr2*), mediators of adhesion (e.g., *S100A8*, *S100A9*, *Sell*, *Selp*, *Thbs1*, *Tnc*, and *Mmp9*), cytokines and their receptors (e.g., *Cd14*, *Csf2rb*, *Csf3r*, *Fpr1*, *Il18rap*, *Il1b*, *Il1f9*, *Il1r2*, *Il1rn*, *Il6*, *Inhba*, *Tnfrsf9*, *Tnfsf14*, *Lilrb4*, *Mefv*, *Osm*), growth factors and their receptors (e.g., *Ereg*, *Fgf23*), coagulation cascade proteins (e.g., *F10*), and heat shock proteins (e.g., *Hspa1a*, *Hspa1b*). Altogether, these data showed a significant upregulation of both CC and CXC subfamilies of chemokine transcripts in the early period of myocardial ischaemia. In addition, data confirmed the upregulation of genes involved in apoptosis as well as apoptosis- and autophagy-related pathways in the ischaemic myocardium, regardless of the underlying genotype (Supplementary Figure 2). Moreover, analyzing the unique 1,428 downregulated genes in STAT1^F77A/F77A^ mice showed a corresponding attenuation of fatty acid oxidation and propanoate metabolism in the cardiac tissue, which may point to a metabolic shift from utilization of fatty acids to other substrates after LAD ligation in the heart. Based on these data, a dysfunctional STAT1 signalling increased the transcription of immune markers including IL-6, triggered immune cell trafficking into the ischaemic heart, and induced metabolic changes, which had an overall beneficial effect on cardiac output in the early stages of MI (Figure 6).

## Discussion

The onset of MI is a complex process driven by several immune cells, cytokines, chemokines and other circulating effector proteins that are recruited to the infarct site in the early stages of ischaemia (4). While the impact of TNF, IL-6 and other cytokines in mediating cardiac inflammation is well characterized, questions on the role of STAT1 oligomerization in the failing heart remain unanswered or, at best, poorly understood. Our study investigated the biological significance of STAT1 in sterile inflammation induced by MI and, in particular, addressed the role of STAT1 co-operative DNA binding in an organismic context. Upon investigating the clinical consequences of an impaired STAT1 signalling in mice after LAD ligation-induced ischaemia, we found that female mice expressing an oligomerisation-deficient STAT1 point mutant exhibited better haemodynamic and cardiac outcomes. Particularly, in the early stages of MI, mice carrying homozygous F77A alleles had an improved LV function in echocardiographic assessment, when compared to their WT littermates. In comparison to the WT group, the end-systolic volume was significantly reduced in the knock-in mice group at day 3 and week 1 after MI. Additionally, measurements of LV fractional area shortening and ejection fraction revealed a better cardiac outcome post-infarction in the STAT1^F77A/F77A^ female mice, which was most pronounced one week after surgery. A beneficial effect of a dysfunctional STAT1 signalling was also observed for male STAT1^F77A/F77A^ mice, which exhibited a lower short-term mortality rate and improved long-term survival compared to WT mice. The marked drop in survival observed after MI at day 5 in our male mice could be due to left ventricular rupture or heart failure, a phenomenon that has been reported to occur between day two and seven after LAD surgery with a 2-3-fold increased incidence in male mice (31).

Sex-related differences in mortality and cardiac function following murine myocardial infarction have been reported in literature with a higher survival rate due to a lower incidence of ventricular rupture in female as compared to male mice (29–34). Despite similar infarct sizes, female mice undergo less extensive left ventricular remodelling than males as measured by means of echocardiography, with reduced dilation and a better-preserved systolic function 28 days after MI (35). One of the several mechanisms behind this sex-based shielding from adverse cardiac remodelling in females could be attributed to the anti-oxidative and anti-apoptotic actions of oestrogen and the expression of oestrogen receptors in the heart (31). Pullen et al. found that female mice showed a more pronounced infiltration with reparative monocytes in the myocardium following coronary ligation as compared to male mice, suggesting that infiltrating monocytes in the infarcted area have an overall protective function (36). This finding is in line with our results showing an increased myocardial infiltration with immune cells is associated with better haemodynamic outcome in the F77A female mice as compared to their WT littermates.

In these experiments, a higher ratio of STAT3 to STAT1 protein levels was detected in the infarcted myocardial tissue of STAT1^F77A/F77A^ animals accompanied by an increase in the distribution of cells expressing STAT1, as compared to their WT littermates. Furthermore, our transcriptome data from early infarcted tissue of WT and STAT1^F77A/F77A^ animals revealed differential upregulation of genes involved in immune response such as chemokines and their receptors in the transgenic animals as compared to WT mice. The increased expression of immune-responsive, inflammatory genes and cell adhesion markers went along with a corresponding downregulation of transcripts involved in mitochondrial fatty acid oxidation and oxidative phosphorylation pathways in the knock-in mice. Notably, STAT1 has been implicated in the Warburg effect in the context of tumorigenesis, due to its modulation of genes involved in glycolysis (37).

Based on these data, we hypothesize that the observed beneficial effect of impaired STAT1 co-operative DNA binding may be driven, at least partially, by the compensatory increase in the level of STAT3, as shown by our Western-blot results. Several studies reported a cardio-protective role of STAT3, in which a lack of STAT3 expression under inflammatory stimuli resulted in cardiomyocyte apoptosis, increased fibrosis, and cardiac dysfunction in aging mice (38). The beneficial effects of STAT3 primarily arise from its transcriptional regulation of stress-induced anti-oxidative genes and the induction of a pro-survival response program (39). Against general opinion that the heart is a post-mitotic organ, several studies have shown that cardiomyocytes can indeed dedifferentiate and re-enter the cell cycle to proliferate, when triggered by hypoxia (40, 41). Combining these observations with a recently discovered role of IFNγ in promoting the differentiation of stem cells into cardiomyocytes (42), the STAT1 amino-terminal deficiency and associated impairment of IFNγ signalling with a corresponding upregulation of STAT3 in our STAT1^F77A/F77A^ mice, may induce dedifferentiation and subsequent proliferation of cardiomyocytes, triggering regeneration in the infarcted myocardium (22). The improved cardiac function in our female STAT1^F77A/F77A^ mice is also in line with other studies that report a detrimental effect of the STAT1-driven apoptotic machinery in cardiomyocytes after ischaemia/reperfusion injury (20, 21, 43). Due to the induction of autophagy by STAT1, STAT1-deficient mice have been found to exhibit a significantly smaller infarct size in an *ex vivo* heart perfusion model (22). However, during the early phase of MI (at day 3), we did not observe significant differences in the infarct size between our STAT1^F77A/F77A^ mice and their WT littermates.

The early onset of MI is characterized by an intense sterile inflammation accompanied by immune cell infiltration, followed by a resolution of inflammation paving the way for wound healing and cardiac repair in late MI (44). Accumulating evidence shows that the inflammatory response is a key factor in myocardial remodelling associated with cytokine and chemokine signalling and the recruitment, migration and adhesion of various subsets of immune cells triggering fibroblast activation and promoting scar formation at the infarct site (2, 45, 46). While several immunomodulatory therapies targeting cardiac inflammation after MI show promising results in pre-clinical and clinical trials, their translational value toward long-term amelioration of cardiac function has not been compelling (47). This discrepancy in the success of anti-inflammatory therapies partly arises from the ambivalent role of inflammation in MI.

The immune cell invasion of the infarcted area in STAT1^F77A/F77A^ mice is reflected by a characteristic pro-inflammatory gene signature profile in this region. While uncontrolled inflammation after ischaemia may lead to adverse cardiac remodelling and heart failure, our study in STAT1 oligomerization-deficient mice after LAD ligation shows that an early increased inflammation can be beneficial for the preservation of cardiac function and the overall survival after acute MI.

The transcriptomic profile of the infarcted myocardium from STAT1^F77A/F77A^ mice as compared to their WT littermates displayed a reduction in the expression of genes involved in mitochondrial oxidative phosphorylation, citrate cycle and cardiac muscle contraction, which are highly expressed in cardiomyocytes. This observation from the RNA-seq data can be due to hypoxia and the presence of infiltrating immune cells at the infarct site, which are known to trigger a metabolic reprogramming for energy production and restoration of homeostasis from fatty acid oxidation as the preferred substrate utilization in the heart to glycolysis (48, 49). The transcriptomic downregulation of components involved in the citrate cycle, oxidative phosphorylation and cardiac muscle contraction observed 24 h after LAD ligation may have driven the improvement in echocardiographic parameters measured at later time points in the STAT1^F77A/F77A^ mouse line.

The pronounced hypoxia-induced, transcriptomic shift from fatty acid oxidation to glycolysis in the infarct area observed in our transgenic mice lacking STAT1 co-operative DNA binding is corroborated by the histological finding of an albeit non-significant increase in the immune cell infiltration of the infarct site. In WT mice, the expression of a fully functional STAT1 protein may induce apoptosis of both immune cells and the surrounding cardiomyocytes in the peri-infarct region, thereby limiting the extent of inflammation. STAT1 is known to upregulate the expression of caspases executing apoptotic cell death and promote fibroblast activation with increased extracellular matrix deposition (2, 42). However, the induction of apoptosis singularly requires the expression of STAT1, as a knock-out showed no apoptosis despite the unaltered apoptotic potential of the wild-type protein or the Y701F mutant defective in tyrosine phosphorylation (23). Similarly, a STAT1 mutant lacking cytokine-induced nuclear accumulation of phospho-STAT1 due to a disrupted nuclear localization signal (NLS) that fails to induce cytokine-mediated gene expression, nevertheless executes apoptosis normally (50). Therefore, the F77A point mutation with abolished co-operative DNA binding may have little impact on the induction of apoptosis in the transgenic mice.

A major limitation of this study results from the confounding of an unbalanced sex distribution in the biochemical and echocardiographic studies. Another limitation includes the unexplored mechanistic role of co-operativity-deficient STAT1 and the correspondingly increased STAT3 expression on cardiomyocyte apoptosis and autophagy in cells after LAD ligation. Similarly, the impact of the F77A mutation in STAT1 and enhanced STAT3 expression on the extent of cardiac fibrosis in the infarct areas of these mice has not been covered in this preliminary investigation, as we focused exclusively on the very early and early phase of acute myocardial infiltration (≤3 days post-surgery). Due to the exploratory nature of this work, our study lacks absolute measurements of acute infarct sizes across the genotypes and a quantitative analysis of immune cell infiltration. Our data in the STAT1^F77A/F77A^ mice showing improved left ventricular function and survival following LAD ligation point to a putative beneficial role of inflammation-induced repair mechanisms in the ischaemic heart.

In summary, defective oligomerization of STAT1 mediated by a lack of reciprocal amino-terminal interactions between adjacent dimers leads to dysfunctional STAT1 signalling and a compensatory increase in STAT3 activation. In the context of experimental MI, a substitution mutation that disrupts tetramer formation triggers early infiltration of immune cells into the infarct area and is associated with beneficial effects on left ventricular remodelling. Further research is needed to unravel the underlying mechanisms for the improved haemodynamic outcome and functional recovery in the ischaemic hearts.

## Data availability statement

The original contributions presented in the study are included in the article/Supplementary material, further inquiries can be directed to the corresponding author.

## Ethics statement

The animal study was reviewed and approved by Niedersächsisches Landesamt für Verbraucherschutz und Lebensmittelsicherheit, 13/1226.

## Author contributions

AD, TR, AI, and TM conceived and designed the study. AD, TR, JS, and PM conducted the experiments. AD, TR, JS, PM, FL, OW, UV, AI, and TM analyzed and discussed the data. AD, TR, PM, JS, and TM wrote the manuscript with contributions of all co-authors. All authors have read and approved the final version of the manuscript.

## Funding

This project was supported by the SFB1002 Service Project (TPS – echocardiography). The research was funded by grants from the Deutsche Forschungsgemeinschaft (DFG) to TM and OW. AD and TR are fellows and AI and TM are principal investigators of the International Research Training Group (IRTG) 1816, funded by the DFG.

## Conflict of interest

The authors declare that the research was conducted in the absence of any commercial or financial relationships that could be construed as a potential conflict of interest.

## Publisher’s note

All claims expressed in this article are solely those of the authors and do not necessarily represent those of their affiliated organizations, or those of the publisher, the editors and the reviewers. Any product that may be evaluated in this article, or claim that may be made by its manufacturer, is not guaranteed or endorsed by the publisher.
