# Supplementary material for: Lack of STAT1 co-operative DNA binding protects against adverse cardiac remodelling in acute myocardial infarction
Source: Front Cardiovasc Med. 2023 Feb 27;10:975012. doi: 10.3389/fcvm.2023.975012 (PMC10008942; doi:10.3389/fcvm.2023.975012)
Supplement: Supplementary file 1 [file Table_1.DOCX]

Supplementary Table 1

|  | Time | Genotype | Mean (CI) | Min | Q1 | Median | Q3 | Max | *p*-value |
| --- | --- | --- | --- | --- | --- | --- | --- | --- | --- |
| End-systolic left ventricular  inner diameter (mm) | **Pre-MI** | **WT** | 3.50 (3.38-3.63) | 3.15 | 3.26 | 3.47 | 3.73 | 4.15 | 0.99 |
|  |  | **STAT1-F77A** | 3.47 (3.34-3.59) | 2.76 | 3.3 | 3.5 | 3.68 | 3.98 |  |
|  | **Day 3** | **WT** | 4.39 (4.15-4.64) | 3.58 | 4.19 | 4.4 | 4.51 | 5.03 | **0.036*** |
|  |  | **STAT1-F77A** | 3.92 (3.59-4.25) | 2.95 | 3.68 | 3.79 | 4.25 | 4.71 |  |
|  | **Week 1** | **WT** | 5.40 (4.6-6.20) | 3.42 | 5.05 | 5.51 | 6.35 | 6.68 | **0.049*** |
|  |  | **STAT1-F77A** | 4.38 (3.7-5.05) | 3.43 | 3.72 | 4.3 | 4.86 | 5.86 |  |
|  | **Week 4** | **WT** | 5.55 (4.26-6.83) | 3.83 | 4.32 | 5.82 | 6.75 | 7.03 | 0.52 |
|  |  | **STAT1-F77A** | 4.94 (3.75-6.13) | 3.4 | 4.03 | 4.64 | 5.35 | 7.94 |  |
| End-diastolic left ventricular inner diameter (mm) | **Pre-MI** | **WT** | 4.29 (4.18-4.4) | 3.83 | 4.14 | 4.25 | 4.39 | 5.01 | 0.52 |
|  |  | **STAT1-F77A** | 4.31 (4.22-4.4) | 3.94 | 4.19 | 4.35 | 4.46 | 4.62 |  |
|  | **Day 3** | **WT** | 4.79 (4.64-4.94) | 4.33 | 4.68 | 4.76 | 4.94 | 5.19 | **0.0035*** |
|  |  | **STAT1-F77A** | 4.39 (4.14-4.64) | 3.46 | 4.25 | 4.56 | 4.62 | 4.82 |  |
|  | **Week 1** | **WT** | 5.60 (5.05-6.16) | 4.36 | 5.33 | 5.66 | 6.09 | 6.86 | 0.061 |
|  |  | **STAT1-F77A** | 4.96 (4.43-5.49) | 4.18 | 4.34 | 5.06 | 5.36 | 5.98 |  |
|  | **Week 4** | **WT** | 5.9 (4.94-6.85) | 4.57 | 5.1 | 5.83 | 6.8 | 7.09 | 0.45 |
|  |  | **STAT1-F77A** | 5.47 (4.52-6.43) | 4.31 | 4.59 | 5.39 | 5.93 | 7.76 |  |
| End-systolic area (mm^2^) | **Pre-MI** | **WT** | 8.28 (7.68-8.88) | 6.43 | 7.34 | 8.2 | 8.98 | 11.96 | 0.38 |
|  |  | **STAT1-F77A** | 7.92 (7.32-8.52) | 5.77 | 6.97 | 7.8 | 8.54 | 11.55 |  |
|  | **Day 3** | **WT** | 12.79 (11.54-14.05) | 9.1 | 11.64 | 12.93 | 14.17 | 15.91 | **0.0068*** |
|  |  | **STAT1-F77A** | 10.24 (8.92-11.55) | 6.64 | 9.27 | 10.09 | 11.11 | 13.27 |  |
|  | **Week 1** | **WT** | 18.09 (13.54-22.65) | 8.76 | 14.42 | 17.14 | 21.62 | 27.96 | **0.030*** |
|  |  | **STAT1-F77A** | 11.55 (7.79-15.31) | 6.17 | 8.83 | 11.7 | 12.22 | 21.12 |  |
|  | **Week 4** | **WT** | 20.64 (12.79-28.50) | 9.18 | 13.38 | 23.19 | 28.01 | 29.35 | 0.38 |
|  |  | **STAT1-F77A** | 16.43 (7.76-25.10) | 6.51 | 9.63 | 14.18 | 18.19 | 39.39 |  |
| End-diastolic area (mm^2^) | **Pre-MI** | **WT** | 12.2 (11.6-12.81) | 10.01 | 11.2 | 12.27 | 12.79 | 15.89 | 0.95 |
|  |  | **STAT1-F77A** | 12.22 (11.55-12.90) | 9.47 | 11.56 | 12.01 | 12.96 | 16.62 |  |
|  | **Day 3** | **WT** | 15.83 (14.81-16.85) | 12.3 | 15.52 | 16.34 | 16.79 | 17.81 | **0.013*** |
|  |  | **STAT1-F77A** | 13.57 (12.17-14.98) | 9 | 12.39 | 13.63 | 15 | 16.49 |  |
|  | **Week 1** | **WT** | 21.29 (16.79-25.79) | 12.7 | 19.4 | 22.02 | 24.2 | 32.24 | 0.061 |
|  |  | **STAT1-F77A** | 15.54 (11.5-19.58) | 10.72 | 12.13 | 15.15 | 16.6 | 25.74 |  |
|  | **Week 4** | **WT** | 24.68 (17.06-32.31) | 12.34 | 18.73 | 25.62 | 32.24 | 32.91 | 0.27 |
|  |  | **STAT1-F77A** | 20.52 (11.86-29.19) | 11.58 | 12.95 | 19.07 | 22.71 | 43.23 |  |
| Epicardial systolic area (mm^2^) | **Pre-MI** | **WT** | 18.19 (17.4-18.98) | 15.93 | 16.73 | 17.34 | 19.23 | 22.66 | 0.29 |
|  |  | **STAT1-F77A** | 18.61 (17.78-19.44) | 14.57 | 17.71 | 18.4 | 19.58 | 23.67 |  |
|  | **Day 3** | **WT** | 25.02 (23.57-26.47) | 20.73 | 23.83 | 24.75 | 26.35 | 28.62 | 0.17 |
|  |  | **STAT1-F77A** | 23.21 (20.18-26.24) | 14.12 | 21.04 | 22.91 | 25.33 | 31.5 |  |
|  | **Week 1** | **WT** | 29.52 (24.32-34.71) | 20.27 | 25.03 | 29.1 | 32.28 | 41.8 | **0.039*** |
|  |  | **STAT1-F77A** | 22.6 (18.36-26.84) | 17.17 | 18.47 | 23.15 | 24.22 | 32.66 |  |
|  | **Week 4** | **WT** | 34.49 (26.27-42.72) | 21.26 | 27.83 | 38.71 | 40.83 | 44.16 | 0.15 |
|  |  | **STAT1-F77A** | 28.97 (17.9-40.04) | 17.07 | 20.87 | 25.86 | 30.56 | 59.27 |  |

|  | Time | Genotype | Mean (CI) | Min | Q1 | Median | Q3 | Max | *p*-value |
| --- | --- | --- | --- | --- | --- | --- | --- | --- | --- |
| Fractional shortening (%) | **Pre-MI** | **WT** | 18.35 (16.43-20.28) | 10.84 | 15.16 | 17.19 | 22.27 | 26.06 | 0.49 |
|  |  | **STAT1-F77A** | 19.54 (17.19-21.90) | 10.74 | 15.84 | 18.14 | 22.84 | 31.27 |  |
|  | **Day 3** | **WT** | 9.47 (5.85-13.08) | 3.11 | 4.81 | 8.56 | 12.98 | 21.21 | 0.56 |
|  |  | **STAT1-F77A** | 10.88 (7.06-14.71) | 2.28 | 7.13 | 12.96 | 14.1 | 18.8 |  |
|  | **Week 1** | **WT** | 6.79 (1.82-11.77) | 0.55 | 2.58 | 4.93 | 9.81 | 21.67 | 0.11 |
|  |  | **STAT1-F77A** | 12.23 (7.50-16.95) | 1.88 | 9.22 | 13.13 | 14.95 | 21.19 |  |
|  | **Week 4** | **WT** | 7.31 (0.02-14.6) | 0.13 | 0.93 | 2.34 | 14.81 | 17.24 | 0.35 |
|  |  | **STAT1-F77A** | 11.38 (6.56-16.19) | 2.34 | 8.13 | 12.38 | 13.84 | 21.07 |  |
| Fractional area shortening (%) | **Pre-MI** | **WT** | 32.38 (29.86-34.91) | 21.25 | 30.93 | 32.38 | 35.73 | 45.46 | 0.22 |
|  |  | **STAT1-F77A** | 35.26 (32.23-38.29) | 18.62 | 32.04 | 33.33 | 39.07 | 49.9 |  |
|  | **Day 3** | **WT** | 19.33 (14.07-24.58) | 8.9 | 10.51 | 19.53 | 26.17 | 30.58 | 0.21 |
|  |  | **STAT1-F77A** | 24.72 (20.16-29.29) | 15.97 | 20.02 | 25.76 | 28.74 | 36.84 |  |
|  | **Week 1** | **WT** | 16.13 (9.7-22.56) | 6.98 | 10.65 | 14.09 | 22.14 | 31.01 | **0.024*** |
|  |  | **STAT1-F77A** | 26.92 (20.36-33.48) | 17.96 | 20.88 | 26.2 | 29.84 | 42.42 |  |
|  | **Week 4** | **WT** | 18.52 (10.15-26.9) | 9.49 | 11.6 | 13.86 | 25.2 | 32.73 | 0.45 |
|  |  | **STAT1-F77A** | 23.29 (14.49-32.08) | 8.89 | 18.18 | 23.62 | 26.43 | 43.81 |  |
| Ejection fraction (%) | **Pre-MI** | **WT** | 37.82 (35.23-40.41) | 25.52 | 35.6 | 37.46 | 42.4 | 49.68 | 0.11 |
|  |  | **STAT1-F77A** | 41.08 (38.23-43.93) | 25.37 | 37.76 | 39.65 | 45.43 | 54.71 |  |
|  | **Day 3** | **WT** | 22.27 (17.45-27.08) | 11.65 | 15.57 | 23.04 | 28.93 | 31.61 | 0.10 |
|  |  | **STAT1-F77A** | 27.39 (22.25-32.54) | 16.9 | 20.91 | 27.53 | 34.71 | 37.89 |  |
|  | **Week 1** | **WT** | 18.60 (11.26-25.95) | 7.84 | 11.14 | 16.59 | 24.23 | 36.59 | **0.030*** |
|  |  | **STAT1-F77A** | 30.11 (22.64-37.57) | 18.69 | 24.41 | 29.05 | 34.08 | 47 |  |
|  | **Week 4** | **WT** | 21.55 (12.64-30.46) | 11.25 | 14.41 | 16.54 | 30.26 | 33.76 | 0.52 |
|  |  | **STAT1-F77A** | 27.36 (17.33-37.38) | 10.31 | 21.11 | 28.32 | 32.31 | 48.46 |  |
| End-systolic volume (mm^3^) | **Pre-MI** | **WT** | 48.50 (44.3-52.7) | 33.58 | 42.47 | 46.68 | 54.67 | 75.21 | 0.25 |
|  |  | **STAT1-F77A** | 45.08 (41.36-48.8) | 32.28 | 40.73 | 44.56 | 50.86 | 64.85 |  |
|  | **Day 3** | **WT** | 79.49 (69.93-89.05) | 55.23 | 71.23 | 81.4 | 85.01 | 108.4 | **0.039*** |
|  |  | **STAT1-F77A** | 62.44 (52.42-72.45) | 39.05 | 54.82 | 60.11 | 69.01 | 86 |  |
|  | **Week 1** | **WT** | 119.69 (84.14-155.23) | 46.72 | 93.47 | 108.07 | 148.48 | 194.47 | **0.039*** |
|  |  | **STAT1-F77A** | 72.00 (42.88-101.12) | 36.61 | 52.32 | 66.16 | 78.59 | 148.88 |  |
|  | **Week 4** | **WT** | 145.13 (83.69-206.57) | 57.03 | 88.86 | 161.71 | 200.87 | 217.71 | 0.27 |
|  |  | **STAT1-F77A** | 108.43 (37.11-179.76) | 34.04 | 58.41 | 75.94 | 128.26 | 299.99 |  |
| End-diastolic volume (mm^3^) | **Pre-MI** | **WT** | 77.69 (72.7-82.68) | 59.02 | 72.24 | 76.89 | 83.34 | 110.86 | 0.78 |
|  |  | **STAT1-F77A** | 76.46 (71.59-81.32) | 56.03 | 70.81 | 76.27 | 81.72 | 106.57 |  |
|  | **Day 3** | **WT** | 101.9 (93.03-110.78) | 75.72 | 94.42 | 104.22 | 112.07 | 124.1 | **0.045*** |
|  |  | **STAT1-F77A** | 85.82 (74.52-97.12) | 53.89 | 75.85 | 83.24 | 97.68 | 107.4 |  |
|  | **Week 1** | **WT** | 143.94 (108.35-179.53) | 73.67 | 125.49 | 142.63 | 167.09 | 228.05 | 0.061 |
|  |  | **STAT1-F77A** | 100.36 (68.71-132.01) | 66.42 | 77.08 | 90.74 | 110.85 | 183.09 |  |
|  | **Week 4** | **WT** | 178.84 (116.48-241.2) | 85.47 | 127.35 | 182.2 | 235.29 | 258.94 | 0.22 |
|  |  | **STAT1-F77A** | 139.31 (66.55-212.07) | 66.05 | 86.83 | 109.76 | 162.51 | 334.46 |  |
