# Supplementary material for: Lack of STAT1 co-operative DNA binding protects against adverse cardiac remodelling in acute myocardial infarction
Source: Front Cardiovasc Med. 2023 Feb 27;10:975012. doi: 10.3389/fcvm.2023.975012 (PMC10008942; doi:10.3389/fcvm.2023.975012)
Supplement: Supplementary file 2 [file Table_2.DOCX]

Supplementary Table 2

| Genotype | Gene | log_2_fold change | Padj* | log_10_padj | baseMean |
| --- | --- | --- | --- | --- | --- |
| Wild-type | *Hspa1a* | 3.77 | 9.56e-11 | 1.00e+01 | 1851.08 |
|  | *Hspa1b* | 3.66 | 4.59e-10 | 9.34e+00 | 2979.38 |
|  | *Fgf23* | 3.65 | 1.91e-10 | 9.72e+00 | 216.89 |
|  | *Cxcl2* | 3.62 | 4.60e-11 | 1.03e+01 | 1167.84 |
|  | *Ngp* | 3.43 | 2.40e-08 | 7.62e+00 | 49.94 |
|  | *Fosb* | 3.39 | 1.53e-08 | 7.82e+00 | 478.94 |
|  | *AA467197* | 3.31 | 4.37e-08 | 7.36e+00 | 690.60 |
|  | *Cxcl10* | 3.28 | 3.62e-09 | 8.44e+00 | 422.35 |
|  | *Ccl4* | 3.27 | 1.02e-09 | 8.99e+00 | 393.55 |
|  | *Cd177* | 3.25 | 1.28e-09 | 8.89e+00 | 406.20 |
|  | *Thbs1* | 3.24 | 5.26e-11 | 1.03e+01 | 12485.02 |
|  | *Hcar2* | 3.23 | 1.46e-08 | 7.84e+00 | 337.96 |
|  | *Cxcl3* | 3.21 | 1.20e-08 | 7.92e+00 | 1422.77 |
|  | *Asprv1* | 3.21 | 6.28e-11 | 1.02e+01 | 290.54 |
|  | *Ptgs2* | 3.18 | 3.82e-08 | 7.42e+00 | 1411.21 |
|  | *Slc7a11* | 3.17 | 1.36e-08 | 7.87e+00 | 422.98 |
|  | *Retnlg* | 3.16 | 1.20e-11 | 1.09e+01 | 797.23 |
|  | *Il6* | 3.16 | 7.46e-09 | 8.13e+00 | 532.09 |
|  | *Selp* | 3.16 | 5.26e-11 | 1.03e+01 | 1177.32 |
|  | *Il1r2* | 3.15 | 1.98e-08 | 7.70e+00 | 1517.34 |
| STAT1-F77A | *Fgf23* | 4.89 | 1.29e-19 | 1.89e+01 | 216.89 |
|  | *Cxcl3* | 4.76 | 9.07e-20 | 1.90e+01 | 1422.77 |
|  | *Slfn4* | 4.64 | 8.59e-21 | 2.01e+01 | 5478.06 |
|  | *Rab44* | 4.52 | 1.37e-17 | 1.69e+01 | 788.41 |
|  | *Cxcl2* | 4.51 | 1.38e-18 | 1.79e+01 | 1167.84 |
|  | *Cd177* | 4.49 | 3.60e-19 | 1.84e+01 | 406.20 |
|  | *Gm5483* | 4.45 | 1.53e-16 | 1.58e+01 | 136.50 |
|  | *Il1r2* | 4.41 | 2.53e-17 | 1.66e+01 | 1517.34 |
|  | *Cd300lf* | 4.40 | 4.25e-22 | 2.14e+01 | 1932.80 |
|  | *Slfn1* | 4.34 | 9.07e-20 | 1.90e+01 | 1233.29 |
|  | *Ptgs2* | 4.27 | 2.51e-15 | 1.46e+01 | 1411.21 |
|  | *Cxcr2* | 4.19 | 3.23e-18 | 1.75e+01 | 1119.46 |
|  | *Slc7a11* | 4.19 | 9.62e-16 | 1.50e+01 | 422.98 |
|  | *Hcar2* | 4.18 | 5.19e-15 | 1.43e+01 | 337.96 |
|  | *Sh2d5* | 4.15 | 1.04e-13 | 1.30e+01 | 925.10 |
|  | *Adamts4* | 4.13 | 5.50e-14 | 1.33e+01 | 3382.60 |
|  | *Trem1* | 4.13 | 2.77e-17 | 1.66e+01 | 631.05 |
|  | *Hspa1a* | 4.12 | 5.50e-14 | 1.33e+01 | 1851.08 |
|  | *Mirt2* | 4.12 | 4.52e-13 | 1.23e+01 | 69.10 |
|  | *Il6* | 4.09 | 1.18e-15 | 1.49e+01 | 532.09 |

*padj: Benjamini-Hochberg adjusted p-value
