# Supplementary material for: Lack of STAT1 co-operative DNA binding protects against adverse cardiac remodelling in acute myocardial infarction
Source: Front Cardiovasc Med. 2023 Feb 27;10:975012. doi: 10.3389/fcvm.2023.975012 (PMC10008942; doi:10.3389/fcvm.2023.975012)
Supplement: Supplementary file 3 [file Table_3.DOCX]

**Supplementary Table 3**

|  | KEGG ID | KEGG pathway (STAT1-F77A MI vs sham) | mean | p value | q value | size |
| --- | --- | --- | --- | --- | --- | --- |
| Immune Response | mmu04060 | Cytokine-cytokine receptor interaction | 8.7 | 6.39e-17 | 1.41e-14 | 204 |
|  | mmu04062 | Chemokine signalling pathway | 5.7 | 1.53e-08 | 5.60e-07 | 167 |
|  | mmu04514 | Cell adhesion molecules (CAMs) | 5.1 | 3.87e-07 | 8.51e-06 | 117 |
|  | mmu04810 | Regulation of actin cytoskeleton | 4.2 | 2.03e-05 | 3.19e-04 | 195 |
|  | mmu04670 | Leukocyte transendothelial migration | 4.1 | 2.71e-05 | 3.97e-04 | 95 |
|  | mmu04510 | Focal adhesion | 2.5 | 6.26e-03 | 3.53e-02 | 192 |
|  | mmu04611 | Platelet activation | 2.2 | 1.40e-02 | 7.18e-02 | 116 |
|  | mmu04520 | Adherens junction | 2.2 | 1.48e-02 | 7.40e-02 | 70 |
|  | mmu04620 | Toll-like receptor signalling pathway | 4.6 | 4.14e-06 | 7.60e-05 | 85 |
|  | mmu04666 | FcγR-mediated phagocytosis | 3.2 | 7.22e-04 | 6.62e-03 | 85 |
|  | mmu04640 | Hematopoietic cell lineage | 6.2 | 2.89e-09 | 1.59e-07 | 84 |
|  | mmu04659 | Th17 cell differentiation | 3.3 | 5.96e-04 | 5.70e-03 | 93 |
|  | mmu04657 | IL-17 signalling pathway | 6.1 | 5.81e-09 | 2.56e-07 | 78 |
|  | mmu04660 | T cell receptor signalling pathway | 3.4 | 3.77e-04 | 3.77e-03 | 93 |
|  | mmu04658 | Th1 and Th2 cell differentiation | 3 | 1.43e-03 | 1.17e-02 | 79 |
|  | mmu04662 | B cell receptor signalling pathway | 3 | 1.70e-03 | 1.29e-02 | 69 |
|  | mmu04650 | Natural killer cell mediated cytotoxicity | 4.4 | 8.80e-06 | 1.49e-04 | 96 |
|  | mmu04672 | Intestinal immune network for IgA production | 3.6 | 2.95e-04 | 3.09e-03 | 34 |
|  | mmu04610 | Complement and coagulation cascades | 3 | 1.69e-03 | 1.29e-02 | 59 |
|  | mmu04612 | Antigen processing and presentation | 4.9 | 1.83e-06 | 3.66e-05 | 62 |
| Signal Transduction | mmu04151 | PI3K-Akt signalling pathway | 3.1 | 1.02e-03 | 8.96e-03 | 306 |
|  | mmu04630 | Jak-STAT signalling pathway | 3.5 | 2.76e-04 | 3.06e-03 | 124 |
|  | mmu04668 | TNF signalling pathway | 5.5 | 5.72e-08 | 1.40e-06 | 108 |
|  | mmu04064 | NF-κB signalling pathway | 5.6 | 3.62e-08 | 1.14e-06 | 89 |
|  | mmu04621 | NOD-like receptor signalling pathway | 5.5 | 4.48e-08 | 1.23e-06 | 146 |
|  | mmu04010 | MAPK signalling pathway | 3.9 | 4.86e-05 | 6.69e-04 | 272 |
|  | mmu04380 | Osteoclast differentiation | 6.3 | 8.57e-10 | 9.43e-08 | 122 |
|  | mmu04066 | HIF-1 signalling pathway | 2.7 | 4.22e-03 | 2.66e-02 | 95 |
|  | mmu04350 | TGF-β signalling pathway | 2.6 | 5.50e-03 | 3.36e-02 | 73 |
|  | mmu04015 | Rap1 signalling pathway | 2.8 | 2.32e-03 | 1.60e-02 | 188 |
|  | mmu04622 | RIG-I-like receptor signalling pathway | 2.8 | 3.34e-03 | 2.22e-02 | 52 |
|  | mmu04390 | Hippo signalling pathway | 2.3 | 1.14e-02 | 6.13e-02 | 138 |
|  | mmu04115 | p53 signalling pathway | 2.5 | 7.40e-03 | 4.07e-02 | 69 |
|  | mmu04623 | Cytosolic DNA-sensing pathway | 3.9 | 1.05e-04 | 1.28e-03 | 46 |
|  | mmu03013 | RNA transport | 2.3 | 1.19e-02 | 6.25e-02 | 150 |
| Cellular Processes | mmu04144 | Endocytosis | 2.9 | 2.05e-03 | 1.50e-02 | 240 |
|  | mmu04110 | Cell cycle | 2.9 | 2.24e-03 | 1.59e-02 | 119 |
|  | mmu04260 | Cardiac muscle contraction | -3.3 | 5.57e-04 | 1.36e-02 | 67 |
|  | mmu04218 | Cellular senescence | 3 | 1.40e-03 | 1.17e-02 | 155 |
|  | mmu04210 | Apoptosis | 3.5 | 2.79e-04 | 3.06e-03 | 131 |
|  | mmu04217 | Necroptosis | 2.2 | 1.52e-02 | 7.44e-02 | 121 |
|  | mmu04145 | Phagosome | 6.1 | 2.03e-09 | 1.49e-07 | 137 |
|  | mmu04146 | Peroxisome | -4.5 | 5.53e-06 | 2.44e-04 | 78 |
|  | mmu03040 | Spliceosome | 2.5 | 5.88e-03 | 3.40e-02 | 127 |
|  | mmu04141 | Protein processing in endoplasmic reticulum | 2.5 | 5.83e-03 | 3.40e-02 | 155 |
|  | mmu04723 | Retrograde endocannabinoid signalling | -3.6 | 2.02e-04 | 5.56e-03 | 122 |
|  | mmu03008 | Ribosome biogenesis in eukaryotes | 3.9 | 9.28e-05 | 1.20e-03 | 72 |
| Metabolism | mmu00190 | Oxidative phosphorylation | -8.1 | 3.66e-14 | 8.06e-12 | 116 |
|  | mmu00020 | Citrate cycle (TCA cycle) | -5.1 | 3.38e-06 | 1.86e-04 | 30 |
|  | mmu01212 | Fatty acid metabolism | -2.8 | 3.56e-03 | 6.02e-02 | 49 |
|  | mmu00071 | Fatty acid degradation | -4.1 | 5.31e-05 | 1.67e-03 | 37 |
|  | mmu00640 | Propanoate metabolism | -5.8 | 3.59e-07 | 2.63e-05 | 29 |
|  | mmu00380 | Tryptophan metabolism | -3 | 2.01e-03 | 4.01e-02 | 33 |
|  | mmu00620 | Pyruvate metabolism | -4.2 | 4.22e-05 | 1.55e-03 | 33 |
|  | mmu00280 | Valine, leucine and isoleucine degradation | -5.4 | 2.58e-07 | 2.63e-05 | 47 |
|  | mmu00532 | Glycosaminoglycan biosynthesis | 2.8 | 4.19e-03 | 2.66e-02 | 20 |
|  | mmu00650 | Butanoate metabolism | -2.8 | 4.39e-03 | 6.90e-02 | 19 |
|  | mmu00630 | Glyoxylate and dicarboxylate metabolism | -2.7 | 5.15e-03 | 7.55e-02 | 28 |
|  | mmu01200 | Carbon metabolism | -2.9 | 1.98e-03 | 4.01e-02 | 106 |
|  | mmu00982 | Drug metabolism - cytochrome P450 | -2.8 | 3.12e-03 | 5.72e-02 | 37 |
|  | mmu03320 | PPAR signalling pathway | -2.6 | 5.49e-03 | 7.55e-02 | 61 |
|  | mmu00980 | Metabolism of xenobiotics by cytochrome P450 | -2.6 | 6.31e-03 | 8.16e-02 | 39 |
