# Supplementary material for: Lack of STAT1 co-operative DNA binding protects against adverse cardiac remodelling in acute myocardial infarction
Source: Front Cardiovasc Med. 2023 Feb 27;10:975012. doi: 10.3389/fcvm.2023.975012 (PMC10008942; doi:10.3389/fcvm.2023.975012)
Supplement: Supplementary file 4 [file Table_4.DOCX]

Supplementary Table 4

| Gene | log_2_ fold change | log_10_ padj * | KEGG pathway |
| --- | --- | --- | --- |
| *Ccl2* | 3.08 | 1.34e+01 | Chemokine signalling pathway |
| *Ccl22* | 3.31 | 1.17e+01 | Chemokine signalling pathway |
| *Ccr1* | 3.38 | 1.40e+01 | Chemokine signalling pathway |
| *Cd14* | 2.97 | 1.14e+01 | MAPK signalling pathway |
| *Csf2rb* | 3.26 | 1.38e+01 | Jak-STAT signalling pathway |
| *Csf3r* | 3.62 | 1.48e+01 | PI3K-Akt signalling pathway |
| *Cxcl10* | 3.76 | 1.23e+01 | RIG-I-like receptor signalling pathway |
| *Cxcl2* | 4.51 | 1.79e+01 | Chemokine signalling pathway |
| *Cxcl3* | 4.76 | 1.90e+01 | Chemokine signalling pathway |
| *Cxcr2* | 4.19 | 1.75e+01 | Endocytosis |
| *Ereg* | 3.71 | 1.18e+01 | PI3K-Akt signalling pathway |
| *F10* | 3.69 | 1.36e+01 | Complement and coagulation cascades |
| *Fgf23* | 4.89 | 1.89e+01 | Ras signalling pathway |
| *Fpr1* | 3.88 | 1.59e+01 | Rap1 signalling pathway |
| *H2-Q10* | 3.69 | 1.37e+01 | Endocytosis |
| *Hspa1a* | 4.12 | 1.33e+01 | Protein processing in endoplasmic reticulum |
| *Hspa1b* | 3.94 | 1.20e+01 | Protein processing in endoplasmic reticulum |
| *Il18rap* | 3.29 | 1.26e+01 | Cytokine-cytokine receptor interaction |
| *Il1b* | 3.89 | 1.66e+01 | Necroptosis |
| *Il1f9* | 3.90 | 1.65e+01 | Cytokine-cytokine receptor interaction |
| *Il1r2* | 4.41 | 1.66e+01 | Hematopoietic cell lineage |
| *Il1rn* | 3.67 | 1.35e+01 | Cytokine-cytokine receptor interaction |
| *Il6* | 4.09 | 1.49e+01 | HIF-1 signalling pathway |
| *Inhba* | 3.87 | 1.24e+01 | TGF-β signalling pathway |
| *Lilrb4* | 2.90 | 1.16e+01 | Osteoclast differentiation |
| *Mefv* | 3.72 | 1.33e+01 | NOD-like receptor signalling pathway |
| *Mmp9* | 3.82 | 1.57e+01 | Leukocyte transendothelial migration |
| *Oas3* | 3.55 | 1.22e+01 | NOD-like receptor signalling pathway |
| *Osm* | 3.96 | 1.44e+01 | PI3K-Akt signalling pathway |
| *Plaur* | 3.69 | 1.42e+01 | Complement and coagulation cascades |
| *Ptgs2* | 4.27 | 1.46e+01 | NF-ĸB signalling pathway |
| *S100a8* | 3.66 | 1.75e+01 | IL-17 signalling pathway |
| *S100a9* | 3.79 | 1.72e+01 | IL-17 signalling pathway |
| *Sell* | 3.44 | 1.45e+01 | Cell adhesion molecules (CAMs) |
| *Selp* | 4.03 | 1.85e+01 | Cell adhesion molecules (CAMs) |
| *Serpine1* | 3.66 | 1.21e+01 | p53 signalling pathway |
| *Thbs1* | 3.82 | 1.58e+01 | Focal adhesion |
| *Timp1* | 2.89 | 1.14e+01 | HIF-1 signalling pathway |
| *Tnc* | 3.66 | 1.34e+01 | Focal adhesion |
| *Tnfrsf9* | 3.95 | 1.16e+01 | Cytokine-cytokine receptor interaction |
| *Tnfsf14* | 3.57 | 1.26e+01 | NF-ĸB signalling pathway |

*padj: Benjamini-Hochberg adjusted p-value
