# Supplementary figures and images for: Lack of STAT1 co-operative DNA binding protects against adverse cardiac remodelling in acute myocardial infarction
Source: Front Cardiovasc Med. 2023 Feb 27;10:975012. doi: 10.3389/fcvm.2023.975012 (PMC10008942; doi:10.3389/fcvm.2023.975012)

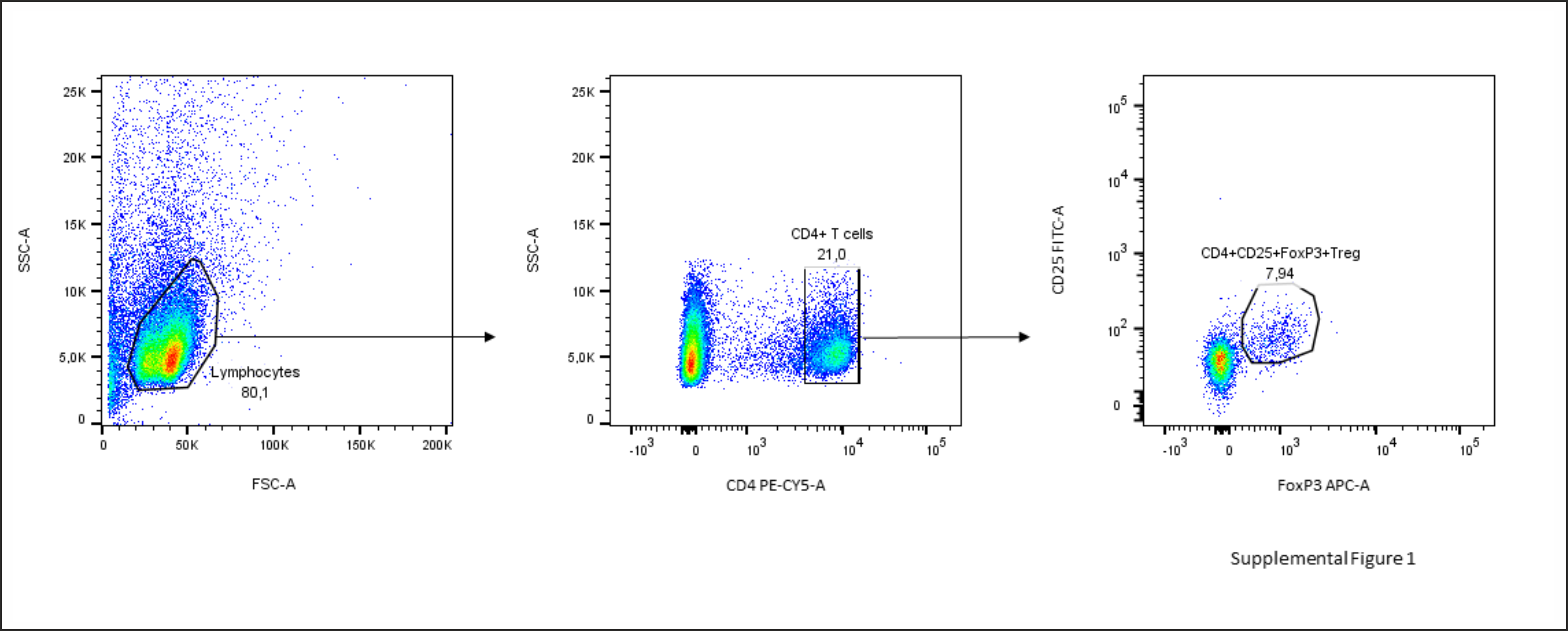

Supplement: Supplementary file 5 [file Image_1.tif]

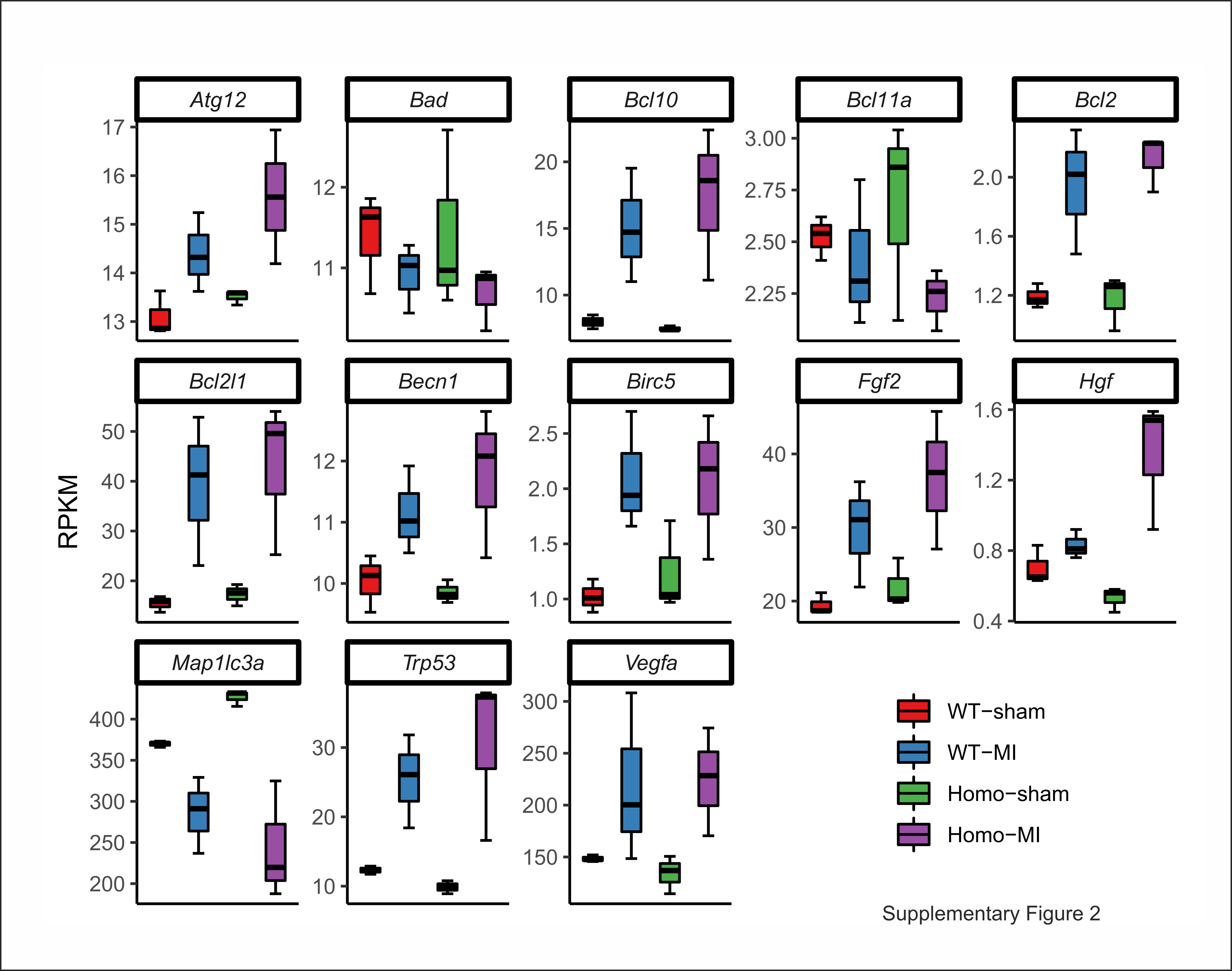

Supplement: Supplementary file 6 [file Image_2.tif]

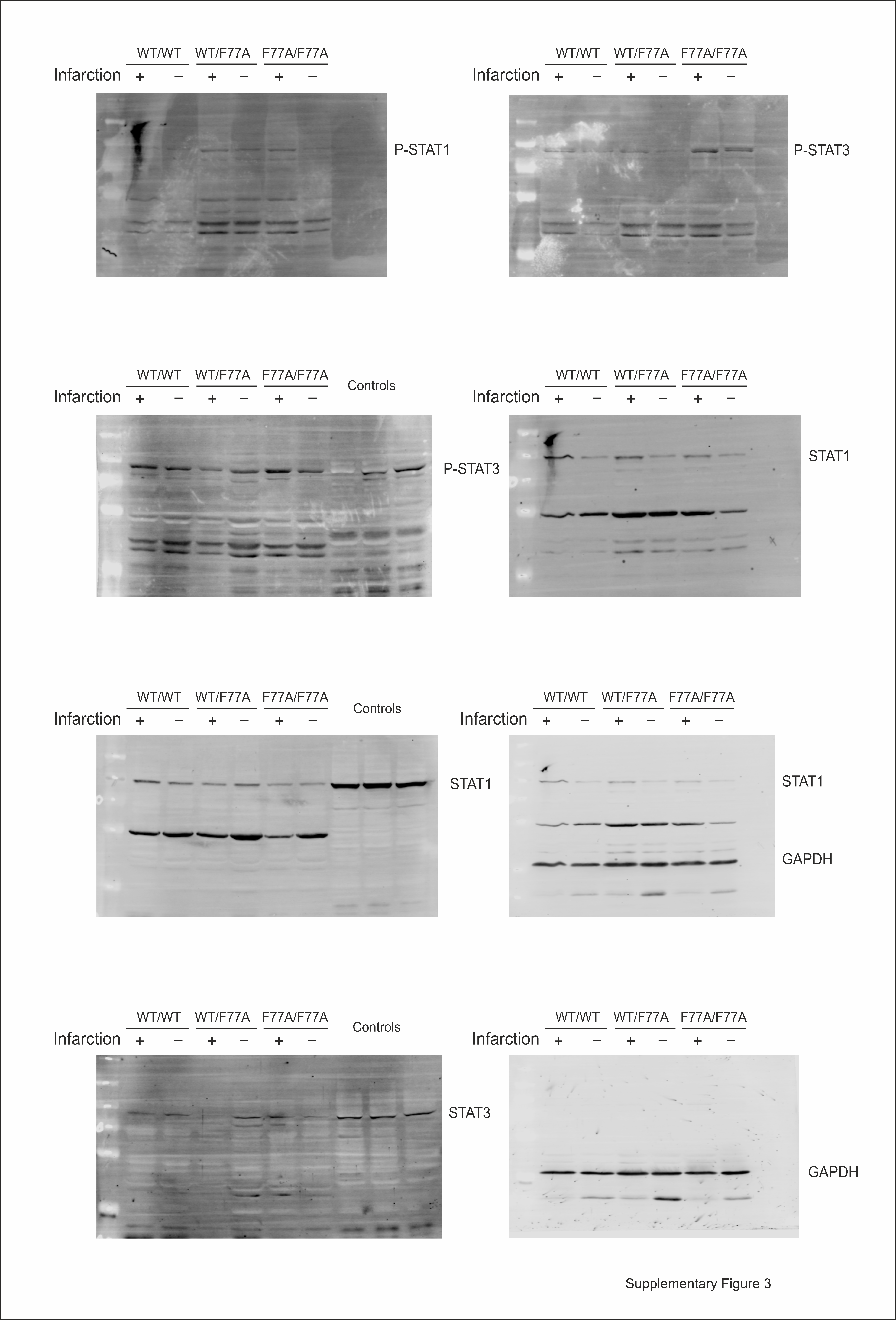

Supplement: Supplementary file 7 [file Image_3.tif]
